# A new species of *Micrurapteryx* (Lepidoptera, Gracillariidae) feeding on *Thermopsislanceolata* (Fabaceae) in southern Siberia and its hymenopterous parasitoids

**DOI:** 10.3897/zookeys.1061.70929

**Published:** 2021-10-08

**Authors:** Natalia I. Kirichenko, Evgeny N. Akulov, Paolo Triberti, Sergey A. Belokobylskij

**Affiliations:** 1 Sukachev Institute of Forest, Siberian Branch of the Russian Academy of Sciences, Federal Research Center ”Krasnoyarsk Science Center SB RAS”, Akademgorodok 50/28, 660036, Krasnoyarsk, Russia Sukachev Institute of Forest, Siberian Branch of the Russian Academy of Sciences Krasnoyarsk Russia; 2 Siberian Federal University, Svobodny pr. 79, 660041, Krasnoyarsk, Russia Siberian Federal University Krasnoyarsk Russia; 3 All-Russian Plant Quarantine Center, Krasnoyarsk branch, Zhelyabova str. 6/6, 660020, Krasnoyarsk, Russia All-Russian Plant Quarantine Center Krasnoyarsk Russia; 4 Museo Civico di Storia Naturale, Lungadige Porta Vittoria 9, I37129, Verona, Italy Museo Civico di Storia Naturale Verona Italy; 5 Zoological Institute of the Russian Academy of Sciences, Universitetskaya nab. 1, 199034, Saint Petersburg, Russia Zoological Institute of the Russian Academy of Sciences St. Petersburg Russia; 6 Museum and Institute of Zoology, Polish Academy of Sciences, 64 Wilcza, Warszawa, 00–679, Poland Polish Academy of Sciences Warsaw Poland

**Keywords:** Biology, DNA barcoding, leaf-mining moth, morphology, new species, parasitoid wasps, pest, the Republic of Khakassia

## Abstract

A new species of leaf-mining moth described here as *Micrurapteryxbaranchikovi* Kirichenko, Akulov & Triberti, **sp. nov.** was detected in large numbers feeding on *Thermopsislanceolata* (Fabaceae) in the Republic of Khakassia (Russia) in 2020. A morphological diagnosis of adults, bionomics and DNA barcoding data of the new species are provided. The developmental stages (larva, pupa, adult), male and female genitalia, as well as the leaf mines and the infestation plot in Khakassia are illustrated; the pest status of the new species in the studied region is discussed. Additionally, parasitism rate was estimated, the parasitoid wasps reared from pupae of the new species were identified (morphologically and genetically) and illustrated . Among them, one ichneumonid, Campoplexsp. aff.borealis (Zetterstedt) and two braconids, *Agathisfuscipennis* (Zetterstedt) and *Illidopssubversor* (Tobias et Kotenko), are novel records for the Republic of Khakassia. Furthermore, they are all documented as parasitoids of Gracillariidae for the first time. The DNA barcode of *A.fuscipennis* is newly obtained and can be used as a reference sequence for species identification.

## Introduction

The genus *Micrurapteryx* Spuler, 1910 (subfam. Ornixolinae Kuznetzov & Baryshnikova, 2001) is a group of leaf-mining micromoths accounting 14 species (Vieira and Karsholt 2010; De Prins and De Prins 2021). The majority are known from some parts of Eurasia: *M.bidentata* Noreika, 1992, *M.bistrigella* (Rebel, 1940), *M.caraganella* (Hering, 1957), *M.fumosella* Kuznetzov & Tristan, 1985, *M.gerasimovi* Ermolaev, 1982, *M.gradatella* (Herrich-Schäffer, 1855), *M.kollariella* (Zeller, 1839), *M.parvula* Amsel, 1935, *M.sophorella* Kuznetzov, 1979, *M.sophorivora* Kuznetzov & Tristan, 1985, *M.tibetiensis* Bai & Li, 2013, and *M.tortuosella* Kuznetzov & Tristan, 1985. The other two species, *M.occulta* Braun, 1922 and *M.salicifoliella* (Chambers, 1872), occur exclusively in North America. No *Micrurapteryx* species has a Holarctic distribution (De Prins and De Prins 2021). All these species were described in 19^th^ and 20^th^ centuries, except *M.tibetiensis* that was described from China in 2013 (Bai 2013) and *M.caraganella* that was redescribed from Siberia (Russia) in 2016 (Kirichenko et al. 2016).

The moths of *Micrurapteryx* are commonly specialised on legumes (Fabaceae), i.e., on *Astragalus*, *Cytisus*, *Genista*, *Laburnum*, *Lathyrus*, *Lupinus*, *Melilotus*, *Sophora*, *Trifolium*, *Vicia* etc. (De Prins and De Prins 2021). As an exception, *M.salicifoliella* feeds on willows *Salix* spp. (Salicaceae) and *M.bistrigella* on *Morellafaya* (Aiton) Wilbur (Myricaceae). For two species, *M.bidentata* described from Kazakhstan and *M.tibetiensis* from China, host plants remain unknown (Noreika and Puplesis 1992; Bai 2013).

To date, no species of *Micrurapteryx* has been documented to feed on *Thermopsis* (Fabaceae) in Eurasia. In 1875, *Gracilaria* [sic] *thermopsella* was described by Chambers from Colorado (USA) (Chambers 1875). A few decades later, the species was transferred to the genus *Parectopa* (Braun 1925; McDunnough 1939; Davis 1983). Further examinations suggested that *Parectopathermopsella* could belong to the genus *Micrurapteryx* (Eiseman 2019). According to Eiseman (2019), Don Davis examined two males reared from *Thermopsis* in the USA, and their genitalia were similar to *M.occulta*. As such, the historic records of *P.thermopsella* could be attributed to *M.occulta* known to feed on several legume genera in North America (Kirichenko et al. 2016). Unfortunately, no type or other specimens of the species are available (according to D. Davis, pers. comm. to J.-F. Landry, Kirichenko et al. 2016) and thus, comparative studies can be done only based on the description given in Chambers (1875).

DNA barcoding of a larva occasionally found in the mine on *Thermopsislanceolata* R. Brown (Fabaceae) in the Republic of Khakassia near the Black Lake field station of Sukachev Institute of Forest SB RAS (Siberia, Russia) in 2019 pointed at the presence of a genetically divergent lineage of *Micrurapteryx*. Therefore, in 2020 intensive sampling was done in this location in order to collect leaf mines and rear moths for species identification.

Using the integrative taxonomy, we describe a new species, *Micrurapteryxbaranchikovi* Kirichenko, Akulov & Triberti, sp. nov., reared from *Thermopsislanceolata* R.Br. (Fabaceae) in the Republic of Khakassia, Russia. We provide morphological data with bionomic notes of the species, highlight diagnostic characteristics to distinguish between *M.baranchikovi* and the North American “*Parectopa*” *thermopsella*, and analyse DNA barcoding data to define the closest relatives and assess the DNA barcoding gap. Furthermore, we estimate the parasitism rate in the dense moth population in Khakassia and identify parasitoid species associated with *M.baranchikovi*. The different developmental stages (larva, pupa, adult), male and female genitalia of *M.baranchikovi*, as well as the adults of parasitoids, are illustrated, and the pest status of the new moth species in the studied region is discussed.

## Materials and methods

### Sampling

**Figure 1.**
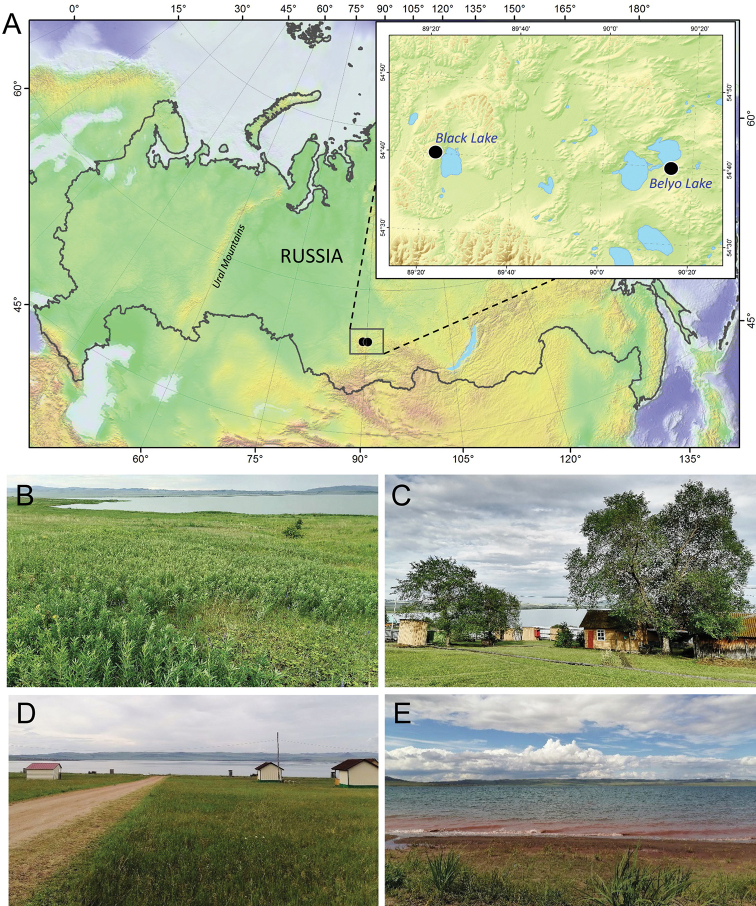
Sampling localities of *Micrurapteryxbaranchikovi* sp. nov. in the Republic of Khakassia, Siberia, Russia **A** map of the sampled area (sampling localities are indicated by black dots) **B** type locality, bank of the Black Lake **C** the Black Lake field station of SIF SB RAS **D, E** bank of the Belyo Lake, beach “Majorca”.

*Thermopsislanceolata* plants with leaf mines of *Micrurapteryxbaranchikovi* sp. nov. were collected in the Republic of Khakassia in two localities in July 2020 (Fig. 1A). A few plants were sampled on the bank of the Belyo Lake (Beljo Ozero) at the beach “Majorca” (Fig. 1B, D) on 6 July 2020, whereas the majority of plants with mines (99% of all plants with mines) were collected between 28–30 July 2020 on the bank of the Black Lake (Chyornoe Ozero), 5 km away from the Black Lake field station of Sukachev Institute of Forest SB RAS (SIF SB RAS) (Fig. 1C, E).

Leaves with young mines sampled in early July were placed in a herbarium; three tiny larvae dissected from the mines were preserved in 95% ethanol. In late July, ca. 50 plants (without roots) were collected and transferred to the insectarium of the Forest zoology laboratory at SIF SB RAS (Krasnoyarsk). Five larvae were dissected from older mines for the ethanol-preserved collection. Plants were placed in bouquets in 5-liter containers filled with water. They were kept in stable conditions (temperature 25 °C, humidity 65%) for one month (until the end of August) to allow larvae to complete their development and pupate. In the containers, water was changed every third day for one month, and the plants were inspected every second day to sample pupating individuals. The cocoons with pupae were cut with a small segment of leaflet and placed by 20 pupae in Petri dishes (90 mm in diameter, 15 dishes in total) lined with filter paper. Overall, 305 pupae were involved in the study, of which five pupae were preserved in 95% ethanol for morphological study.

Pupae were kept under laboratory conditions until September 1^st^, 2020. As no adult emerged from several-week-old pupae, we suspected that the pupae entered diapause and allowed them to overwinter. For that, the dishes with pupae were placed in the fridge (temperature +3 °C): half of the dishes (7 out of 15) on September 1^st^ and the remaining 8 dishes on September 20^th^; the latter dishes were kept under room conditions longer in case some moths would emerge in the first half of September. The dishes were returned to laboratory conditions (temperature 23 °C, humidity 70%) after two months, i.e., the first batch of dishes was taken out from the fridge on November 1^st^ and the second on December 20^th^. The dishes were monitored every day to collect emerged adult moths and parasitoids. Adults were pinned and stored in a dry collection (53 adult moths), parasitoid adults were preserved in 95% ethanol for further morphological and molecular genetic analyses.

### DNA barcoding

DNA barcoding of *Micrurapteryxbaranchikovi* sp. nov. was performed to define its genetic relatedness to other species of *Micrurapteryx* and assess the DNA barcoding gap (the difference between the largest intraspecific and the smallest interspecific distances). In total, seven specimens of *M.baranchikovi* were involved in the genetic analysis (Suppl. material 1: Table S1); one larva (collected in 2019, as mentioned in the Introduction), two larvae and four adults (collected in 2020). DNA was extracted from whole bodies of larvae and hind legs of adults and sequenced at the Canadian Centre for DNA Barcoding (CCDB, Biodiversity Institute of Ontario, University of Guelph, Ontario, Canada) using primer set C_LepFolF/C_LepFolR, following the standard high-throughput protocol (de Waard et al. 2008). The same protocol was applied for sequencing three specimens of parasitoid wasps: two Ichneumonidae (sample IDs NK-20-32 ♀, NK-20-33 ♂) and one Braconidae (sample ID NK-20-34 ♀) that emerged from pupae of *M.baranchikovi*. The hind legs of parasitoids were used for DNA barcoding; the vouchers were saved for morphological identification. The specimen data of all sequenced material are given in Suppl. material 1: Table S1.

The sequences, trace files, biogeographic data and photographs of the vouchers were deposited in the Barcode of Life Data Systems (BOLD) (Ratnasingham and Hebert 2007; www.barcodinglife.org); the original sequences were also submitted to NCBI (National Centre for Biotechnology Information) to obtain GenBank accessions. All data are publicly available in BOLD (dx.doi.org/10.5883/DS-MICKHA).

For phylogenetic analysis, 38 sequences of five other *Micrurapteryx* species, showing close genetic and/or morphological relatedness to the new species, were involved in the analysis: three from Eurasia, *M.caraganella* (21 sequences), *M.gradatella* (eight), *M.kollariella* (three), and two species from North America: *M.occulta* (three sequences) and *M.salicifoliella* (three) (Suppl. material 1: Table S1). The majority of specimens of those species were sequenced for a previous study (Kirichenko et al. 2016), and some specimens of *M.salicifoliella* were DNA barcoded and published by Hebert et al. (2016).

For analysing genetic distances of parasitoids associated with *M.baranchikovi*, we additionally borrowed eight public sequences of the three species from BOLD: *Campoplexmulticinctus* (six sequences), *C.borealis* (one) and *Agathis* sp. (one) (Suppl. material 1: Table S1).

Barcode Index Numbers (BINs) were retrieved for each species in BOLD (Ratnasingham and Hebert 2013). The sequences were aligned in BioEdit 7.2.5 (Jeanmougin et al. 1998). Maximum likelihood (ML) trees were constructed in MEGA X (Kumar et al. 2018) using Kimura 2-parameter model and bootstrap analysis (1000 iterations). Intra- and interspecific genetic distances were estimated using the same model.

The DNA barcodes of *Parectopaononidis* (Zeller, 1839) (Lepidoptera: Gracillariidae) and *Metallusalbipes* (Cameron, 1875) (Hymenoptera: Tenthredinidae), earlier obtained by NK (Suppl. material 1: Table S1), were used for rooting the respective phylogenetic trees.

### Morphology

The external morphology of *Micrurapteryxbaranchikovi* was examined based on 51 adult specimens. Thirteen adults (three males and ten females) were dissected; genitalia dissections and slide mounts were prepared following Robinson (1976). For comparison, the drawing of the male genitalia of *M.sophorivora* was reproduced from the paper by Kuznetzov and Tristan (1985). For genitalia, the terminology follows Klots (1970) and Kristensen (2003).

The braconid parasitoids reared from the pupae of *M.baranchikovi* were identified based on their morphological characters using the following keys: Tobias et al. (1986), Kotenko (2007), Simbolotti and van Achterberg (1999).

### Imaging

The sampling localities and mined leaves were photographed by NK and EN using a digital camera Sony Nex3; the images of larvae, pupae and pinned adults of the moth were taken by the same camera through a Zeiss Stemi DV4 stereo microscope (Sukachev Institute of Forest SB RAS, Krasnoyarsk, Russia). Genitalia were photographed by PT with a Leica DFC 450 digital camera through Leitz Diaplan GMBH microscope (Museo Civico di Storia Naturale, Verona, Italy) and by EN and NK using a Canon EOS 650D mounted on Olympus CX41 microscope. The parasitoids were photographed by SAB using Olympus OM-D E-M1 digital camera mounted on Olympus SZX10 microscope (Zoological Institute RAS, Saint Petersburg, Russia). All digital photographs were edited and assembled into plates in Adobe Photoshop CS5 Extended.

### Specimen depositories

**SIF** Sukachev Institute of Forest, Siberian Branch of the Russian Academy of Sciences, Krasnoyarsk, Russia (47 pinned moths, ethanol preserved larvae and pupae of the moth, 20 pressed leaves with the mines in herbarium);

**MSNV** Museo Civico di Storia Naturale, Verona, Italy (four pinned moths);

**ZISP** Zoological Institute of the Russian Academy of Sciences, Saint Petersburg, Russia (two pinned moths, 27 pinned parasitoid adults).

## Results

### Molecular data

**Figure 2.**
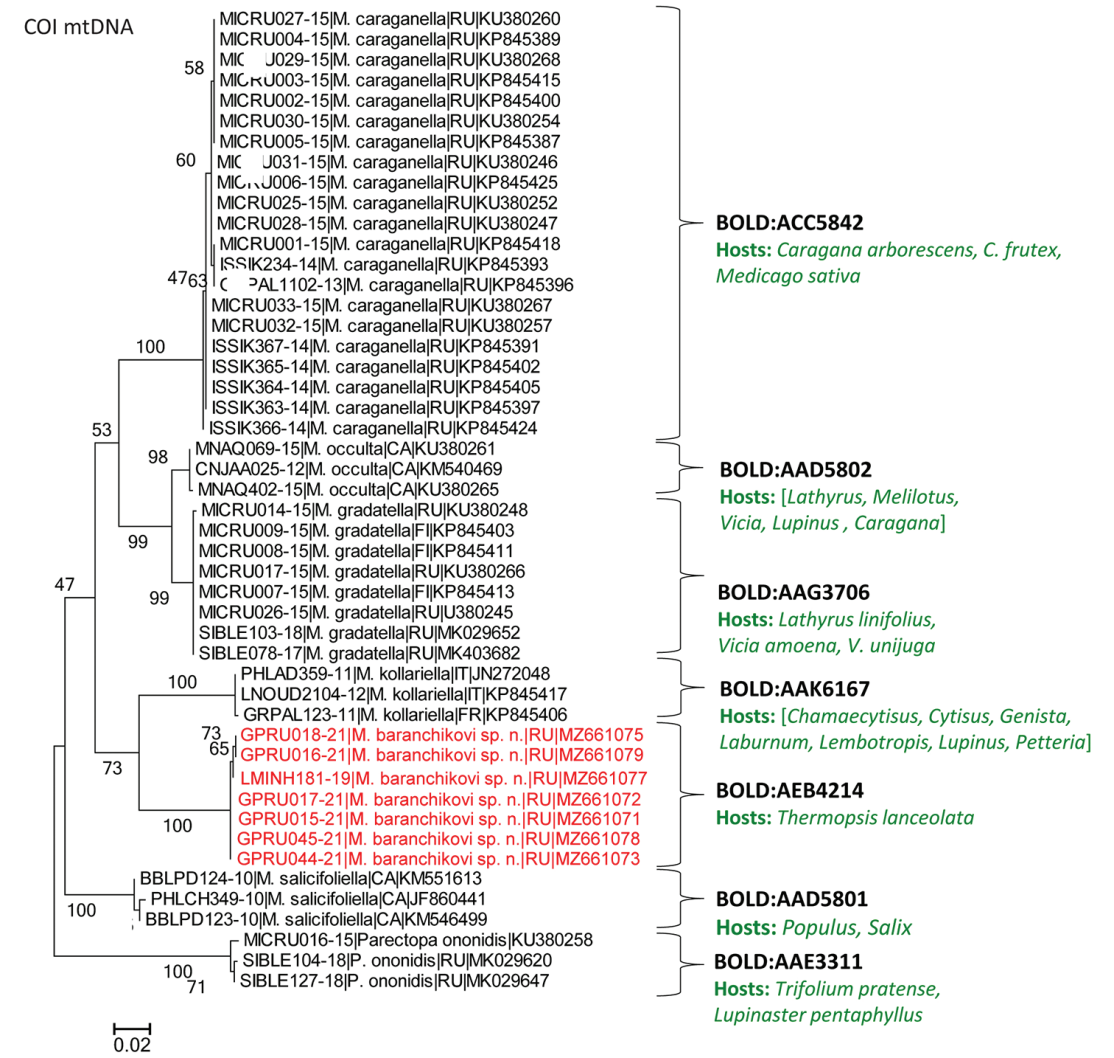
Maximum Likelihood tree showing the proximity of COI barcode sequences of *Micrurapteryxbaranchikovi* sp. nov. (indicated in red) to other *Micrurapteryx* species. Each specimen is indicated by the BOLD process ID, followed by species name, country (CA Canada, FI Finland, FR France, IT Italy, RU Russia), and the GenBank accession number. Bootstrap values are indicated next to the corresponding branches. BIN numbers are given next to each cluster. The host plants are indicated in green; for the cases with no host plant species recorded, the list of host plant genera is provided in square parentheses (as per De Prins and De Prins 2021).

***Micrurapteryx* spp.** The maximum likelihood tree based on COI sequences shows six clusters with 98–100 bootstrap support that match up with Barcode Index Numbers (BINs) (Fig. 2).

In the genus *Micrurapteryx*, the difference between the maximal intraspecific divergence (0.93% in *M.occulta*) and the minimal interspecific divergence (2.17% between *M.gradatella* and *M.occulta*) (Table 1) resulted in a DNA barcoding gap of 1.24%. Assigned to a unique BIN (BOLD:AEB4214), *Micrurapteryxbaranchikovi* showed relatively low intraspecific variability (0.31%; N = 7) (Fig. 2, Table 1). Furthermore, it exhibited a pronounced DNA barcoding gap of 8.99% (the difference between the maximal intraspecific divergence in *M.baranchikovi*, 0.31%, and the minimal interspecific divergence in *M.baranchikovi* – *M.kollariella*, 9.30%; see Table 1).

**Table 1.** Intra- and interspecific divergences in COI mtDNA gene among *Micrurapteryx* spp. Minimal pairwise distances are given for each species pair; values in square brackets represent maximal intraspecific distances.

Species	*Micrurapteryx*					
	*baranchikovi* sp. nov.	*kollariella*	*gradatella*	*caraganella*	*occulta*	*salicifoliella*
*Micrurapteryxbaranchikovi* sp. nov.	[0.31]					
*M.kollariella*	9.30	[0.16]				
*M.gradatella*	10.23	10.08	[0.16]			
*M.caraganella*	10.23	11.01	8.53	[0.62]		
*M.occulta*	10.70	10.54	2.17	7.91	[0.93]	
*M.salicifoliella*	11.32	10.85	8.53	10.08	7.91	[0.47]

*Micrurapteryxkollariella* is the nearest neighbour to *M.M.baranchikovi*, with 59 diagnostic substitutions in the barcode fragment, as based on one studied population of *M.baranchikovi* in Khakassia (Suppl. material 2: Table S2). Notably, the North American *M.salicifoliella*, whose female genitalia have a high similarity to those of *M.baranchikovi* (see the analysis in Morphological section), formed the most distant genetic cluster with a minimal interspecific distance of 11.32% between these two species (Fig. 2, Table 1).

The specimens of the North American *M.occulta*, that might represent a *Thermopsis*-feeding *Micrurapteryx* originally described as *Gracilaria* [sic] *P.thermopsella* by Chambers in the USA (see the taxonomic note in Kirichenko et al. 2016), formed a distant cluster with a minimal interspecific distance of 10.70% to *M.baranchikovi* (Fig. 2, Table 1). In contrast, *M.occulta* sequences showed high similarity to the Eurasian *M.gradatella*, with a minimum genetic distance of 2.17% (Table 1); both species are known to attack *Lathyrus* in the continents where they occur.

**Hymenopterous parasitoids.** By DNA barcoding, the three sequenced specimens of parasitoids were identified as belonging to the two genera: *Campoplex* (Ichneumonidae: Campopleginae) (two specimens) and *Agathis* (Braconidae: Agathidinae) (one specimen). Sequences of two Khakassian specimens of *Campoplex* were identical (Suppl. material 3: Table S3).

**Figure 3.**
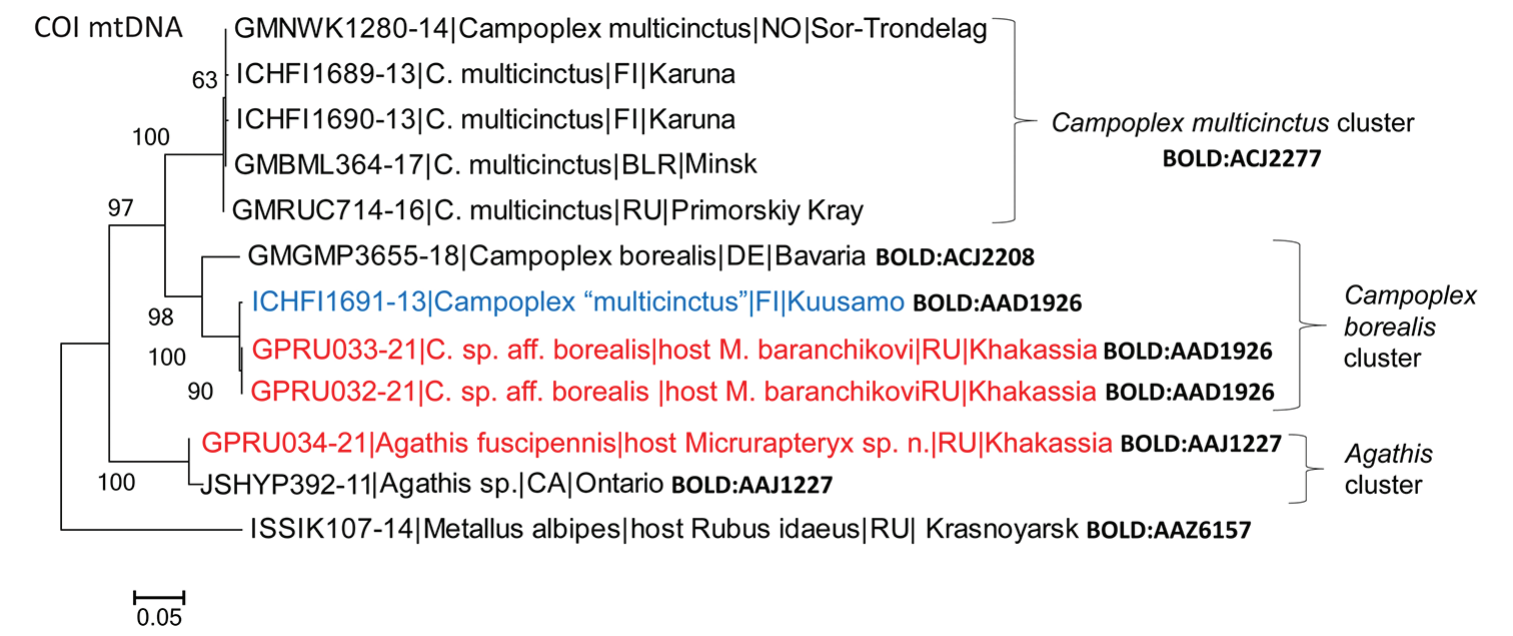
Maximum Likelihood tree showing the proximity of COI barcode sequences of the two hymenopteran parasitoids of *Micrurapteryxbaranchikovi* sp. nov. (indicated in red) to the closest relatives in BOLD. As the specimen identity in BOLD (indicated in blue) is doubtful, the species name is given between quotes. Each specimen is indicated by the BOLD Process ID, followed by species name, host (where known), country (BLR Belarus, CA Canada, DE Germany, FI Finland, NO Norway, RU Russia), and sampling region. Bootstrap values are indicated next to the corresponding branches. BIN numbers are given in bold next to the clusters; if several BINs are known to one species, they are listed next to each specimen.

In BOLD, the Khakassian Campoplexsp. aff.borealis showed little genetic divergence (0.39%) from *C.multicinctus* Gravenhorst sampled in Finland (process ID: ICHFI1691-13, BIN: BOLD:AAD1926) (Fig. 3, Suppl. material 1: Table S1). However, the latter seemed a clear misidentification as it joined a presumable cluster of *Campoplexborealis* in the analysed tree (Fig. 3). Furthermore, Campoplexsp. aff.borealis showed large genetic divergence (11.61%) from the cluster formed by five *C.multicinctus* from Finland, Belarus and Russia assigned to the BIN (BOLD:ACJ2277) (Fig. 3, Suppl. material 3: Table S3).

The genetic distance between the Khakassian Campoplexsp. aff.borealis and the only publicly available DNA barcode of morphologically related *Campoplexborealis* (from Germany, Process ID GMGMP3655-18, A. Hausman coll., BIN: BOLD:ACJ2208) reached 7.16% (Fig. 3; Suppl. material 3: Table S3).

The only sequenced Khakassian specimen of a braconid wasp (NK-20-34 ♀, GPRU034-21) was identified in NCBI only to the genus level (97.92%), with the nearest neighbour *Agathis* sp. from Canada (Process ID JSHYP392-11, BIN: BOLD:AAJ1227; published in Hebert et al. (2016)) (Fig. 3). By morphology, we identified the braconid from Khakassia as *Agathisfuscipennis* (Zetterstedt, 1838). This species has been DNA barcoded for the first time.

In our study, no specimens of *Illidopssubversor* (Tobias & Kotenko, 1986) (Braconidae: Microgastrinae) was DNA barcoded; the species was identified solely by morphology (see below).

### New species taxonomy and biology

#### Family Gracillariidae Stainton, 1854

**Subfamily Ornixolinae Kuznetzov & Baryshnikova, 2001**

##### Genus *Micrurapteryx* Spuler, 1910

###### Micrurapteryx baranchikovi

Taxon classificationAnimaliaLepidopteraGracillarioidea

Kirichenko, Akulov & Triberti sp. nov.

E1F46ED7-1FAF-5AF8-AB00-F2C0C96F063E

http://zoobank.org/C30483F1-DA35-4BB8-983E-B79CB72FD9ED

####### Type material.

**Figure 4.**
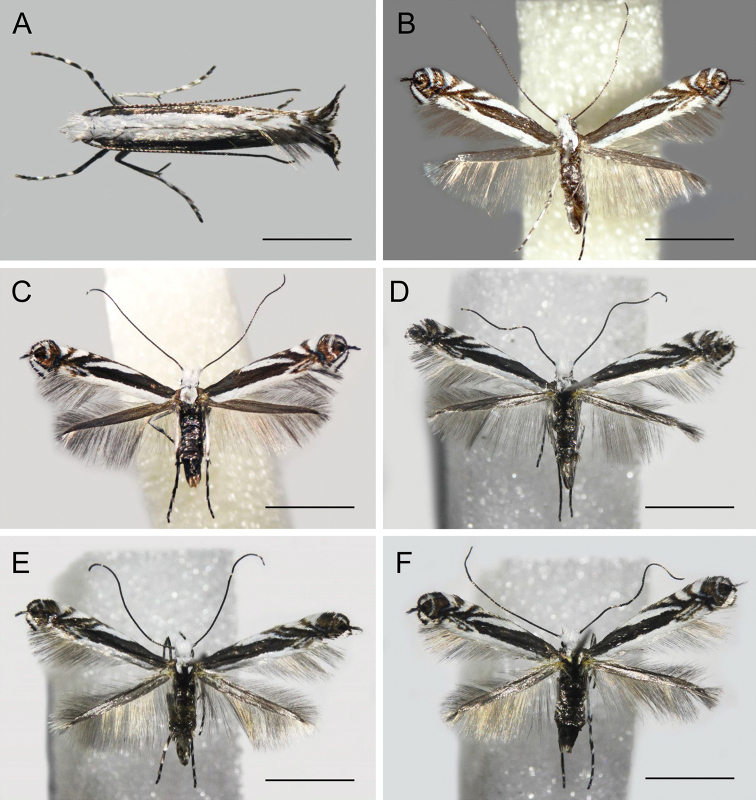
Adults of *Micrurapteryxbaranchikovi* sp. nov. **A** resting posture **B** holotype, field no. NK-08.12-1 (♂), DNA barcoded, Process ID: GPRU015-21**C** paratype, field no. NK-29.11-1 (♀), DNA barcoded, Process ID: GPRU016-21**D** paratype, field no. NK-10-3 (♂) **E** paratype, field no. NK-11-3 (♂) **F** paratype, field no. NK-9-2 (♀). Scale bars: 2.5 mm.

***Holotype*** ♂ (Fig. 4A, B): Republic of Khakassia, near the Black Lake field station of SIF SB RAS, along the lake bank, *Thermopsislanceolata*; 28.VII.2020 coll. (mine), N. Kirichenko & E. Akulov coll, 08.XII.2020 emerged (hereafter indicated as em.), field no. NK-08.12-1 (♂), genitalia slide NK-08.12-1♂, DNA barcoded (Process ID: GPRU015-21) (SIF). ***Paratypes*.** 15♂, 35♀ (Fig. 4C–F). Republic of Khakassia, near the Black Lake field station of SIF SB RAS, along the lake bank, *Thermopsislanceolata*, 28.VII.2020 coll. (mine), Kirichenko N. coll., 14.I.2021 em., field no. NK-11-3, genitalia slide TRB4429♂ (MSNV); same label, but 17.I.2021 em. (from 2 mines), field nos NK-9-2, NK-10-3, genitalia slide TRB4425♀, TRB4428♂ (MSNV); 18.I.2021 em., field no. NK-11-4, genitalia slide TRB4430♀ (MSNV); 27.VII.2020 coll. (2 mines), 22.I.2021 em. 2♂, field nos NK-13-1 (♂), NK-13-3 (♂) (SIF); same label, but N. Kirichenko & E. Akulov coll., 28.VII.2020 coll. (mine), 26.XI.2020 em., field no. NK-26.11-1 (♀), genitalia slide NK-26.11-1♀ (SIF), DNA barcoded (Process ID: GPRU018-21); same label but 08.XII.2020 em., field no. NK-08.12-2 (♂), genitalia slide NK-08.12-2♀ (SIF), DNA barcoded (Process ID: GPRU017-21); same label but 29.XI.2020 em., field no. NK-29.11-1 (♀), genitalia slide NK-29.11-1♀, (SIF), DNA barcoded (Process ID: GPRU016-21); same label but 27.VII.2020 coll. (mine), 7.I.2021 em. 2♀, field nos NK-13-2 (♀), NK-10-1 (♀); same label but 28.VII.2020 coll. (mine), 24.XII.2020 em., field no. NK-11-1 (♀); 30.XII.2020 em., field no. NK-12-1 (♀);same label but 2.I.2021 em. 2♀, field nos NK-16-1 (♀), NK-11-5(♀); same label but 3.I.2021 em., 2♀, field nos NK-9-1 (♀), NK-12-3 (♀), genitalia slide NK-12-3♀; same label but 15.I.2021 em., field no. NK-10-2 (♀); same label but 19.I.2021 em., field no. NK-10-4 (♀); same label but 28.VII.2020 coll. (mine), 14.XI.2020 em. 4♀ & 1♂, field nos NK-14.11-1 (♀), NK-14.11-2 (♀), NK-14.11-3 (♀), NK-14.11-5 (♀), NK-14.11-4 (♂), genitalia slides NK-14.11-1♀, NK-14.11-2♀; same label but 26.XI.2020 em., field no. NK-08.12-3 (♀); same label but 3.XII.2020 em., field no. NK-03.12-1 (♀), genitalia slide NK-03.12-1♀; same label but 7.XII.2020 em. 3♀ & 1♂, field nos NK-07.12-1 (♀), NK-07.12-2 (♀), NK-07.12-3 (♀), NK-07.12-4(♂), genitalia slide NK-07.12-4♂; same label but 8.XII.2020 em., field no. NK-08.12-3 (♂); 15.XII.2020 em. 1♀ & 2♂, field nos NK-15.12-1 (♀), NK-15.12-2 (♂), NK-15.12-3 (♂); same label but 17.XII.2020 em. 3♀ & 1♂, field nos NK-17.12-1 (♀), NK-17.12-2 (♀), NK-17.12-3 (♀), NK-17.12-4 (♂); same label but 20.XII.2020 em. 2♂ & 2♀, field nos NK-20.12-1 (♂), NK-20.12-3 (♂), NK-20.12-2 (♀), NK-20.12-4 (♀), genitalia slide NK-20.12-2♀; same label but 25.XII.2020 em. 1♀ & 1♂, field nos NK-25.12-1 (♀), NK-25.12-2 (♂); same label but 26.XII.2020 em. 1♂ & 2♀, field nos NK-26.12-1 (♂), NK-26.12-2 (♀), NK-26.12-3 (♀), genitalia slide NK-26.12-3♀; 27.XII.2020 em. 1♂ & 2♀, field no. NK-27.12-1 (♂) (SIF).

####### Additional material examined.

Pupa (2): Republic of Khakassia, near the Black Lake field station SIF SB RAS, along the lake bank, *Thermopsislanceolata*, 28.VII.2020 coll. (2 mines), Kirichenko N. coll., field nos NK-28-1, NK-28-2. Larva (4): same label, (mine), filed no. Kh-NK-20-1, DNA barcoded (Process ID: GPRU044-21); same label but 27.VII.2020 coll. (2 mines), field nos NK-27-1, NK-27-2; same republic but Belyo Lake, along the lake bank, 7.VII.2020 coll. (mine), Kirichenko N. coll., filed no. Kh-NK-20-2, DNA barcoded (Process ID: GPRU045-21).

####### Diagnosis

**Figure 5.**
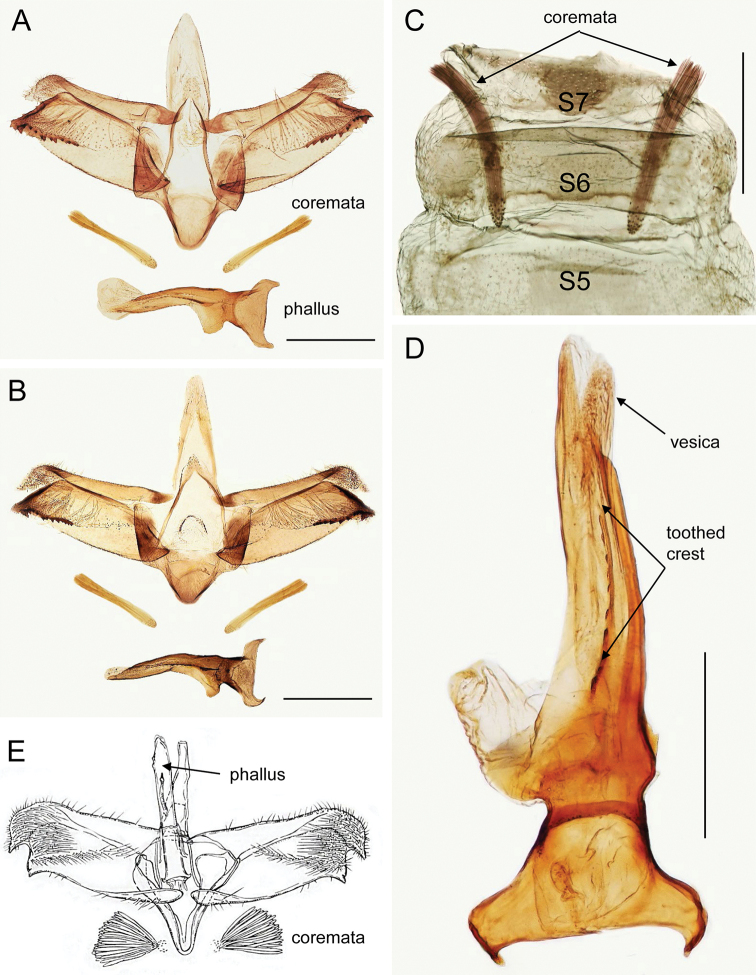
Male genitalia of *Micrurapteryxbaranchikovi* sp. nov. **A** holotype, field no. NK-08.12-1 (♂) genitalia slide NK-08.12-1♂, DNA barcoded (Process ID: GPRU015-21) **B** paratype, field no. NK-07.12-4 (♂), genitalia slide NK-07.12-4♂ **C** paratype, field no. NK-11-3, genitalia slide TRB4428, coremata position in the abdominal segments (S) **D** paratype, field no. NK-11-3, genitalia slide TRB4428, zoomed phallus **E** male genitalia of *Micrurapteryxsophorivora* (drawing reproduced from Kuznetzov and Tristan (1985) with permission of the journal editor). Scale bars: 250 µm (**A–D**).

**(Figs 4, 5).** The forewing pattern of *M.baranchikovi* reflects the typical habits of the genus: a series of costal strigulae, a white band along the dorsal margin and a projection of the fringe line at apex (Fig. 4B–F). However, the genital structures allow easy identification. The male genitalia of *M.baranchikovi* are distinguished from congeners by the pointed and not rounded valvar tip (Fig. 5A, B). This character is present only in *M.sophorivora* Kuznetzov & Tristan, 1985 (Fig. 5E), which is widely distributed in central western Asia and whose larvae feed on *Sophora* and *Robinia* (Fabaceae) (Seven and Genҫer 2009; De Prins and De Prins 2021). The two species are separable by the following characters in the male genitalia: (1) different inclination of the valvar and saccular apices with respect to the horizontal axis of the valvae, at 90° in *M.sophorivora* versus at ca. 45° in *M.baranchikovi*; (2) straight phallus with a single and elongate cornutus, coremata wider and shorter than half phallus in *M.sophorivora* versus somewhat curved phallus, no cornuti, coremata longer than half phallus and thin in *M.baranchikovi* (Fig. 5A–E).

**Figure 6.**
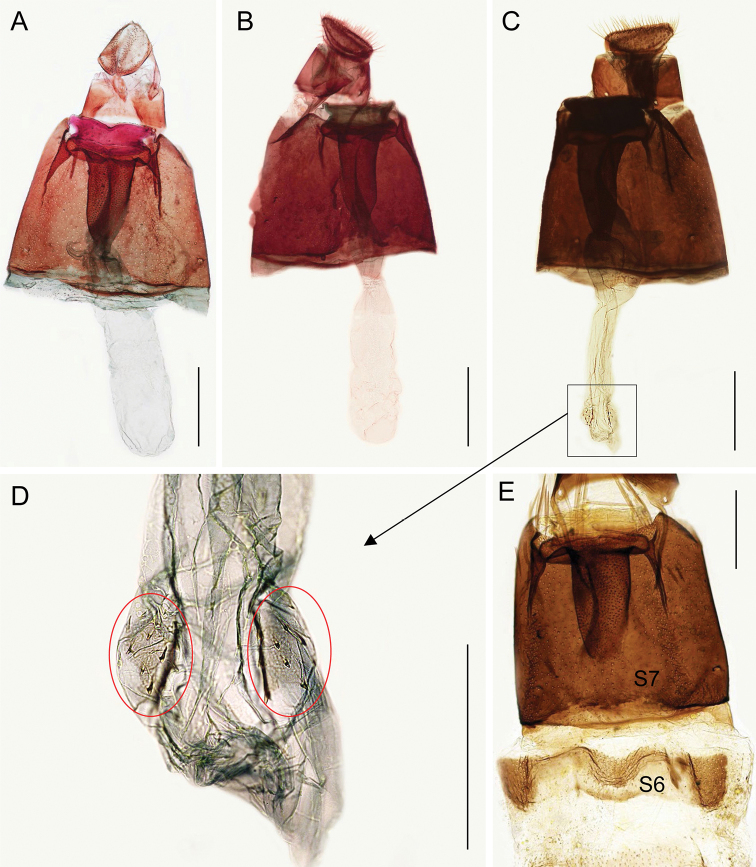
Female genitalia of *Micrurapteryxbaranchikovi* sp. nov. **A** field no. NK-9-2 (♀), genitalia slide TRB4425 **B** field no. NK-08.12-2 (♀), genitalia slide NK-08.12-2♀, DNA barcoded (Process ID: GPRU017-21) **C** field no. NK-29.11-1 (♀), genitalia slide NK-29.11-1♀, DNA barcoded (Process ID: GPRU016-21) **D** enlarged part of bursa copulatrix with two fields of microspines (shown by red circles) **E** segments (S) 6 and 7. Scale bars 200 μm.

In the female genitalia, the differences are the following: thin ductus bursae and piriform corpus bursae with a group of thorn-like signa in *M.sophorivora*, while in *M.baranchikovi* ductus and corpus bursae are not differentiated and signa mostly absent or reduced to ca. ten microspines (Fig. 6A, D). These characters are present in the female genitalia of *M.salicifoliella*, but this species is easy distinguishable by the sclerotised section of antrum / ductus bursae protruding from the anterior margin of segment 7 (S7) (Kirichenko et al. 2016). The male genitalia of *M.baranchikovi* are very different from those of *M.salicifoliella* (see figs 28–29 in Kirichenko et al. 2016).

####### Description of adult

**(Figs 4–6).** Male and female. Alar expanse 8.0–11.0 mm (51 specimens).

***Head*.** Frons, vertex and palpi white with intermixture of dark scales around eyes. Labial palpus rather long and slender, slightly upturned; maxillary palpus ca. half of apical article of labial palpus. Antenna fuscous dorsally, scape, pedicel and ¼ of flagellum white ventrally, remaining articles ringed with paler colour; pecten absent.

***Thorax*.** Dorsum white, ventral side and tegulae brownish grey. Legs white, fore, mid coxae and femurs dark brown outwardly, tibiae and tarsi annulated and of the same colour. Wing venation as in *M.kollariella* (see Vári 1961). Forewing dark brown in ground colour with white markings; costal margin with five white strigulae. First three strigulae almost parallel, oblique and bent outwards. First strigula very dilated on the costal margin and projected backwards, second often obsolescent, last two semi-circular, often both touching opposite margin or, in some specimens, fused apically. Fifth strigula with a dark apical dot. Dorsal margin white in basal 4/5 with two thin, linear projections distally, sometimes not connected to white margin. Cilia white around apex to tornus with dark brown tips interrupted by linear marking protruding from fringe line (not from dark apical dot). Hindwing grey ochreous, cilia pale grey.

***Abdomen*.** Entirely brownish grey, last segments white ventrally, wider in male compared to female. A pair of thin coremata in the intersegmental membrane S5/S6, ca. half the width of S6 (Fig. 5C). S8 weakly sclerotised, tergum reduced to thin, narrow transverse band. In the female S6 shorter than or equal to preceding one and ca. a quarter of S7 long, sternum sclerotised, anterior margin with slight medial convexity (Fig. 6F).

***Male genitalia*.** Tegumen short, subtriangular at apex, with long and thin pedunculi; tuba analis long and membranous, produced beyond tegumen, without suscaphium but with a pair of lateral lamellae, with no setae. Valva longitudinally cleft, costal region slightly concave, apex of cucullus pointed and inclined 45° with respect to longitudinal axis of valva; sacculus markedly developed, rectangular, apex produced into a pointed process with toothed margins, downward-oriented and almost parallel to cucullus (Fig. 5A, B). Phallus ca. 0.9 times length of valva, flattened, base bifurcate, longitudinally a thin, mid-ventral toothed crest and a long, lateral thickening ending before a pointed apex, no cornuti (Fig. 5A, B, D).

***Female genitalia*.** Posterior apophyses not spine-shaped but lamellar, anterior ones longer, linear, and thin. S8 short, ca. same length as posterior apophysis, weakly sclerotised. S7 ca. four times S8 long, sternum markedly sclerotised, elongate subrectangular, its posterior margin modified in a membranous sector provided with a row of long and thin scales. This structure, supported by a sclerotised transverse bar, delimits a wide sinus vaginalis where, ventrally, the ostium bursae opens; opening of ostium is ca. half the width of S7 (Fig. 6E). Antrum and posterior section of ductus bursae undifferentiated, with dorsal wall strongly sclerotised for a length of just over half of S7 and covered with microspinules while the ventral one, membranous, bears a thin longitudinal thickening. Inception of ductus seminalis at anterior end of sclerotised section. Ductus and corpus bursae undifferentiated, signa mostly absent or reduced to ca. ten microspines. Ductus spermathecae with efferent canal forming three coils before vesicle (not shown).

####### Variability

**(Fig. 6).** The new species exhibits considerable diversity in the structures of the bursa copulatrix. In most examined specimens (seven out of ten), ductus and corpus bursae are undifferentiated (Fig. 6A), in three other specimens, the ductus is narrower than corpus bursa (Fig. 6B, C). It could be due to individual variability; another explanation could be related to the method of dissection, in particular boiling, resulting in contraction of ductus in its apical part.

Another issue are microspines in the corpus bursae that were recorded in ca. half of all dissected females. In those females, two opposite weakly sclerotised plates containing ca. ten microspines were present, often hardly visible (Fig. 6C, D). Nevertheless, the adults with such variability did not differ in other morphological characters. The two females were DNA barcoded: one without microspines and another one with microspines in the corpus bursae (Fig. 6B, C); they showed very low genetic divergence (0.3%), leaving no doubt that they are one species.

####### Taxonomic remarks.

Since it is still uncertain whether there is a separate *Thermopsis*-feeding species in North America “”, here we examine the possibility that it could be conspecific with *M.baranchikovi*. To do this, we compared the Chambers’s original description of *thermopsella* (Chambers 1875) with that of *M.baranchikovi*. In the genus *Micrurapteryx*, habitus is quite uniform and indistinguishable for many species (Kirichenko et al. 2016); therefore, any particular character can be important for species differentiation. We detected specific characters in adult morphology allowing to distinguish between “*Parectopa*” *thermopsella* and *M.baranchikovi*. These characters are listed below, with the original description of *thermopsella*’s characters italicised and provided word-by-word as it appeared in Chambers (1875), followed by the indication of differences in *M.baranchikovi*:

(1) “*Outer surface of the second joint of the palpi dark gray brown, inner surface white, third joint whitish with a brownish annulus before the tip.*” In *M.baranchikovi*, labial palpi entirely white.

(2) “*Antennae dark gray brown annulated with white.*” In *M.baranchikovi*, antennae with scape, pedicel and part of flagellum white ventrally, remaining articles ringed with paler colour.

(3) “*The dark brown of the disc is divided into three distinct spots by three short white streaks emitted from the white dorsal margin, and which pass a little obliquely backwards, the first placed before the middle, the second about the middle and the third behind it.*” These markings are absent in the new species.

(4) “*In the grayish part of the wing are five white costal streaks; the first of these is long and narrow, beginning about the basal third of the wing length and passing obliquely backwards until it almost touches the white of the dorsal margin in the apical part of the wing, the second is wider and much shorter, the third shorter and narrower than the second, but both oblique; while the fourth is still shorter, and is nearly perpendicular to the margin.*” In *M.baranchikovi*, the first streak is very dilated on the costal margin and projected backwards, while the third and fourth are well developed and almost similar, always wider than the second, which is almost obsolescent.

####### Larva.

**Figure 7.**
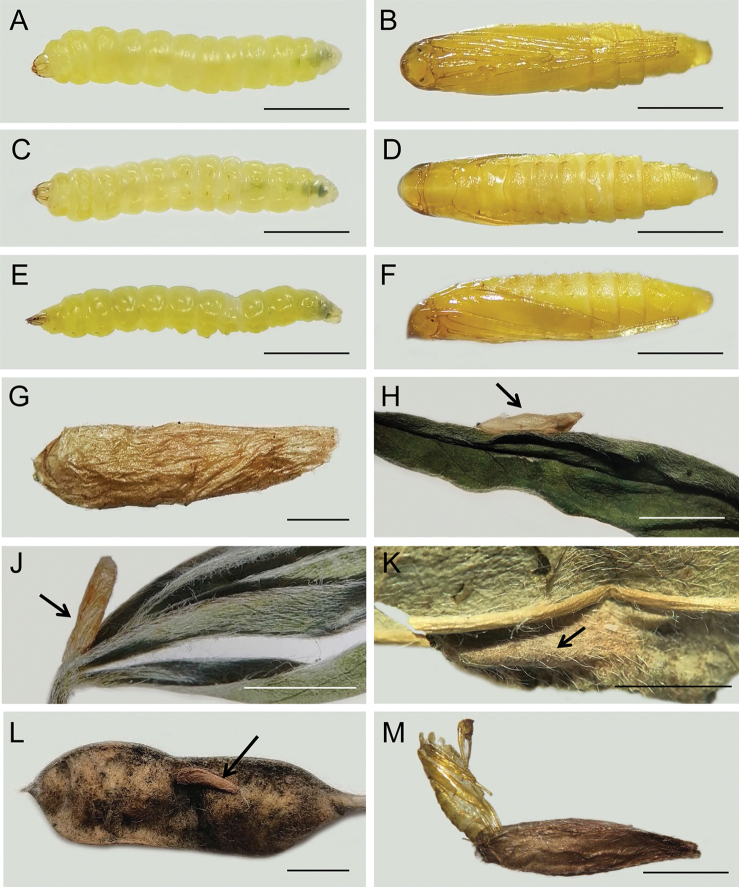
Premature stages of *Micrurapteryxbaranchikovi* sp. nov. and pupation sites **A, C, E** mature larva before pupation [dorsal (**A**), ventral (**C**), lateral (**E**)] **B, D, F** pupa in few a hours after pupation [dorsal (**B**), ventral (**D**), lateral (**F**)] **G** cocoon **H** most common pupation site on the lower side of the leaf next to/or along the midrib. Rare pupation sites: **J** on the basis of the leaf **K** inside the leaf mine along the midrib **L** on legume surface **M** pupal exuviae protruding from cocoon. Arrows show the cocoon. Scale bars: 1.5 mm (**A–F**), 1.2 mm (**G**), 7 mm (**H–L**), 2.5 mm (**M**).

(Fig. 7A, C, E). The studied last instar larvae are tissue-feeders. The larva is yellow. It probably develops in five instars. The morphology does not show particular differences from *M.caraganella* (Kirichenko et al. 2016).

####### Pupa.

**Figure 8.**
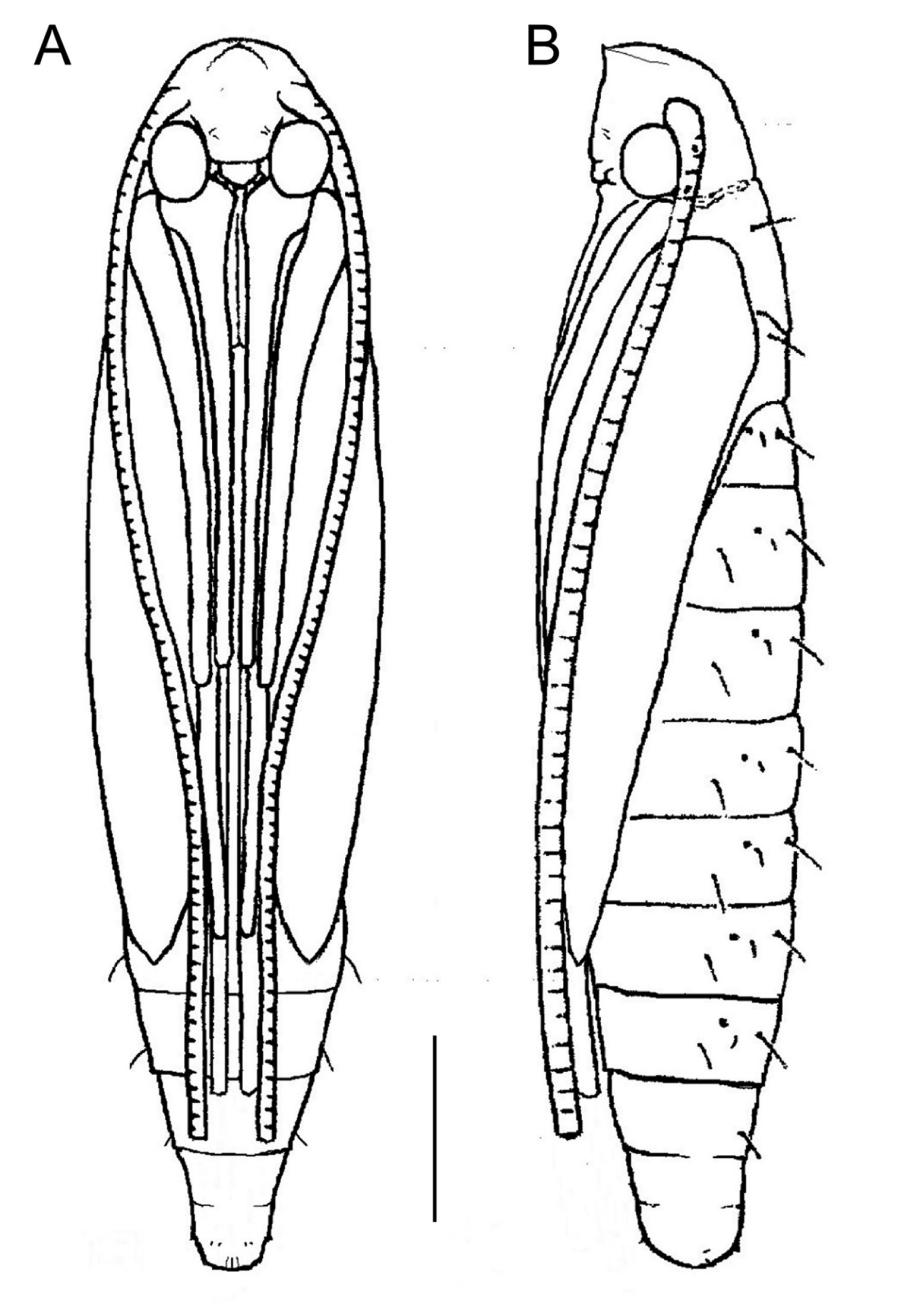
Pupa of *Micrurapteryxbaranchikovi* sp. nov. **A** ventral view **B** lateral view. Scale bar: 0.8 mm.

(Figs 7B, D, F, 8). The young pupa is yellow, soon turns brownish; length 4.5–5.0 mm, width 0.7–1.0 mm. Frontal process (cocoon cutter) simple with pointed projection (Fig. 8); clypeal setae paired, very reduced and nearly contiguous; antenna and hindleg extended to S8; forewing to S5. Setae D1, L1 and SD1 present on S1–S7. Cremaster is very similar to *M.caraganella* (Kirichenko et al. 2016) consisting of a ring of five pairs of small spines, dorsal pair slightly enlarged, the two most ventral pairs are the smallest.

####### Etymology.

The species is named in honour of Dr. Yuri N. Baranchikov, Russian forest entomologist and scientific supervisor of NK and EA, in recognition of his research in regional Lepidoptera and his effective 30-year heading the Black Lake field station of the V.N. Sukachev Institute of Forest SB RAS in Khakassia, around which the new *Micrurapteryx* species was discovered.

####### Bionomics

**Figure 9.**
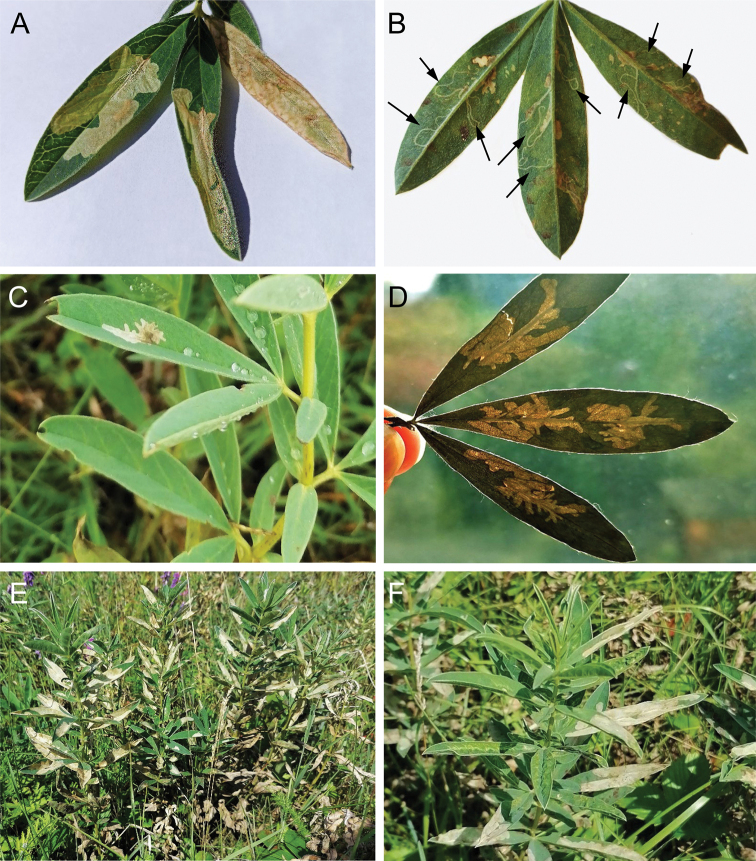
Leaf mines of *Micrurapteryxbaranchikovi* on *Thermopsislanceolata*, the Black Lake field station of SIF SB RAS, Khakassia, Russia **A** upper side blotch mines covering significant part of the leaflets **B** early mines (long narrow tunnels on the lower side of the leaf, the individual mines are indicated by arrows) **C** upper side branched blotch mine **D** multiple upper side branched mines in translucent light **E, F** damaged plants (with large whitish blotch mines on the leaves).

**(Figs 7G–M, 9).** In Khakassia, *M.baranchikovi* develops in one generation annually. Adults are on wing in late May to early June. Oviposition takes place from early to mid-June. Eggs are laid on the lower surface of the leaves. Early instar larvae are found in the mines in late June to early July; late instar larvae in late July to early August. Pupation from early to late August; the pupae hibernate.

The mine (Fig. 9A) is similar to that of other *Micrurapteryx* species (Kirichenko et al. 2016). It starts on a lower side of the leaf (rarely on stipules) as a relatively long contorted well-visible tunnel in the epidermis (Fig. 9B). Soon the larva continues its development in a roundish or slightly branched blotch mine situated above the midrib of a leaflet (Fig. 9C, D). Older mines can occupy almost the complete leaflet (Fig. 9A). If several mines occur per leaflet, enlarging blotch mines merge and several larvae are found in one mine. Initially blotch mines are pale green, later yellowish or whitish. Severely damaged plants turn white (Fig. 9E, F); damaged leaves with abandoned mines soon turn brown and desiccate.

The blotch mines are nearly free of frass; larvae eject frass grains outside the mine by protruding the rear part of the body through a coarse slit that they gnaw in the lower epidermis. Often, frass can be found next to the slit; a few frass grains can be still seen inside the mines. Older larvae are able to vacate the mine and start a new one on a neighbouring leaflet or leaf by making a cut in the lower epidermis of leaf lamina and rapidly biting into the mesophyll.

Pupation is external. The larva vacates the mine and moves to a neighbour or a distant leaf, where it spins a relatively thick cocoon (Fig. 7G). Most often pupation takes place on the lower side of the leaf lamina along the midrib (Fig. 7H). The colour and the shape of the cocoon resembles the midrib, making it difficult to spot the cocoon. In dense populations, the pupation may occur in other places: on the upper side of the leaf next to the leaf edge, in the basis of the leaf (Fig. 7J), exceptionally inside the mine (Fig. 7K) or on a legume surface (Fig. 7L). The thick cocoon seems to help pupae to overwinter in the steppe with little or no snow cover. After adult emergence, the pupal exuviae (i.e., the 2/3 of its length) protrude through the opening of the cocoon (the least dense part of the cocoon) (Fig. 7M). In 2020, under laboratory conditions, the adults started emerging in ca. two weeks after they were moved from the fridge (where they overwintered for two month at a temperature of +3 °C) to room conditions (temperature + 23 °C, humidity 70%), and the emergence lasted for ca. one month.

####### Host plant.

The host plant is the perennial legume herb, the lanceolate bush-pea, *Thermopsislanceolata* (Fabaceae). The name *T.lanceolata* was proposed in 1811 by R. Brown for the plant from Siberia which was mistakenly determined in 1803 by P. Pallas under the name of *Sophoralupinoides* L. (Zhu and Kirkbride 2005). *Thermopsislanceolata* grows in the steppe with chernozemic solonetzic and sandy soil, on stony and gravelly slopes, in meadows and agricultural fields, along lakes and rivers, as well as on disturbed areas and in/around settlements (Tolmachev 1974; Telyatiev 1985).

The plant is poisonous due to the high concentration of alkaloids (Telyatiev 1985). However, these alkaloids also make it a medical herb (Volynskiy et al. 1978). In Russia, the antitussive drugs produced from *T.lanceolata* have been utilised for decades to treat tracheitis, bronchitis and pneumonia (Telyatiev 1985; Lager 1988; Vidal Handbook 2021). Furthermore, the alcaloid cytisine extracted from *T.lanceolata* is a major component of respiratory analeptic drag against asphyxia (Telyatiev 1985). Finally, this alkaloid is also used for treating nicotine addiction (Telyatiev 1985; West et al. 2011; Hajek et al. 2013). *Thermopsislanceolata* was reported to be weedy in some parts of Russia (Telyatiev 1985). Getting into hay, it can poison livestock, in particular horses (Telyatiev 1985; Minaeva 1991).

####### Distribution.

The leaf mines of *Micrurapteryxbaranchikovi* were found in the steppe area of the Republic of Khakassia (south-western Siberia) in two localities: around Black Lake (in 5 km from the Black Lake field station of SIF SB RA) and next to Belyo Lake (beach “Majorca”). The sampling area is situated in a temperate climatic zone (Grigoryev and Budyko 1960). Summers are dry and hot, with a number of sunny days in the republic, higher than in neighbouring regions; winters are cold, with little snow. The average air temperature in July is +18 °C, in January −19 °C (Samoilova et al. 2019). In the steppe area, the average annual precipitation is ca. 250 mm per year; up to 70% of precipitation falls in summer, of which 55% falls in August with rains and showers (Samoilova et al. 2019). The growing season lasts ca. 165 days (Samoilova et al. 2019).

It is highly likely that the moth is distributed across most of the republic where the host plant is present. Also, bearing in mind that *T.lanceolata* has rather extensive range in the Urals, in some other parts of Western and Eastern Siberia, as well as in Central Asia (Kazakhstan, Kyrgyzstan), Northern China and Mongolia (Kotunkov 1974; Telyatiev 1985; Lager 1988; Wu and Raven 2010), the occurrence of the moth in these regions is quite possible.

####### Outbreak character.

In July-August 2020, we recorded a local outbreak of the moth in the type locality, covering an area of ca. 500 m^2^. Up to 15 mines per leaf and up to 57 mines per plant were documented. The damage peak occurred in late July to mid-August, i.e., when the leaf mines reached their maximal size (and some still contained mature larvae) and the abandoned mines turned brown and dried out. Bearing in mind that *T.lanceolata* is used in Russia for medical purpose, severe damage caused by the new species may locally affect its harvesting in the Republic of Khakassia.

####### Indoor survival rate.

In 2020, we obtained 53 moths from 300 pupae by indoor rearing; the survival rate of *Micrurapteryxbaranchikovi* (at the pupal stage) was only 17.6% (53/300). Twenty seven out of 247 pupae were parasitised (see next section). The remaining 220 pupae (73.3%) did not succeed to develop to adults after hibernation.

####### Parasitoids.

Overall, 27 parasitoid adults emerged from 300 pupae of *M.baranchikovi*, i.e., parasitism level was only 9% in 2020. Under laboratory conditions, the emergence of parasitoids lasted 12 days, from 10^th^ to 21^st^ of August.

The reared parasitoids were identified to three taxa: two braconids, *Agathisfuscipennis* (Zetterstedt, 1838) (Agathidinae) and *Illidopssubversor* (Tobias & Kotenko, 1986) (Microgastrinae), and one ichneumonid Campoplexsp. aff.borealis (Zetterstedt, 1838) (Campopleginae). The identification of the first two species was done by external morphology; *A.fuscipennis* was additionally DNA barcoded, however, by its DNA barcode it was determined to genus level only. We failed to provide an exact identification of the Khakassian *Campoplex* neither by morphology nor by DNA barcoding. Morphologically, the examined specimens showed similarity to *Campoplexborealis* by colour of hind legs and shape of temple in dorsal view (see taxonomic note below).

All parasitoids reared from *M.baranchikovi* are solitary species. In our laboratory rearing, *A.fuscipennis* dominated and accounted for 18 out of 27 parasitoids (i.e., 67% of all emerged parasitoids), followed by Campoplexsp. aff.borealis (6 adults, 22%) and *I.subversor* (3 adults, 11%). They all represented novel records for the Republic of Khakassia and were documented as parasitoids of Gracillariidae for the first time.

#### Order Hymenoptera Linnaeus, 1758

**Family Braconidae Nees, 1811**

**Subfamily Agathidinae Haliday, 1833**

##### Genus *Agathis* Latreille, 1804

###### Agathis fuscipennis

Taxon classificationAnimaliaHymenopteraIchneumonoidea

(Zetterstedt, 1838)

4C7B7E55-3B0A-5A9D-BC33-385C3B334DD5

####### Material examined.

1♀, 1♂, Republic of Khakassia, near Black Lake field station of SIF SB RAS, 27.VII.2020 coll. (mine), 11–12.VIII.2020 em., Kirichenko N. coll, no. BL-14-20, host: *Micrurapteryx* sp. nov. (pupa), plant: *Thermopsislanceolata*; Kirichenko N. det.; 1♀, 1♂, same labels, but 11–13.VIII.2020 em., no. BL-13-20; 1♀, same labels, but 27.VI.2020 coll. (mine), 17.VIII.2020 em., no. BL-16-20; 2♀, same labels, but 28.VII.2020 coll. (mine), 13–17.VIII.2020 em., no. BL-11-20; 1♂, same labels, but 28.VII.2020 coll. (mine), 14.VIII.2020 em., no. BL-1-20-2; 2♂, same labels, but 28.VII.2020 coll. (mine), 16–18.VIII.2020 em., no. BL-12-20; 1♀, 1♂, same labels, but 28.VII.2020 coll. (mine), 18–21.VII.2020 em., no. BL-9-20; 1♀, same labels, but VII.2020 coll. (mine), 17–19.VIII.2020 em. no. BL-8-20-1 (legs taken for DNA); 3♂, same labels, but 28.VII.2020 coll. (mine), 14–20.VIII.2020 em., no. BL-10-20; 2♂, same labels, but VII.2020 coll. (mine), 11–16.VIII.2020 em., no. BL-7-20-2. All deposited in ZISP.

**Figure 10.**
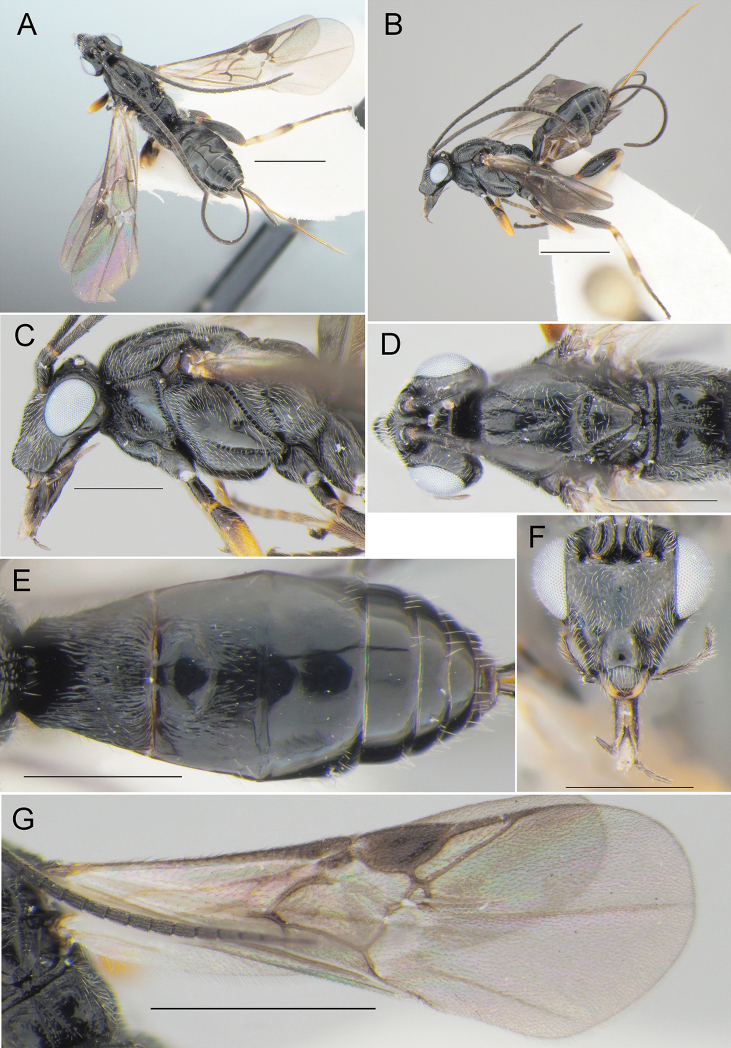
*Agathisfuscipennis* (Zetterstedt, 1838) (♀, reared specimen, 27.VII.2020 (mine) coll., 11-12.VIII.2020 em., no. BL-13-20, host: *Micrurapteryxbaranchikovi* sp. nov., the Republic of Khakassia, Russia) **A** habitus, dorsal view **B** habitus, lateral view **C** head and mesosoma, lateral view **D** head and mesosoma, dorsal view **E** metasoma, dorsal view **F** head, front view **G** fore wing. Scale bars: 1 mm (**A, B, G**), 0.5 mm (**C–F**).

####### Hosts.

(from Yu et al. 2016). **Lepidoptera, Coleophoridae**: *Coleophoraalbicostella* (Duponchel, 1842) [on *Potentilla* sp.: Rosaceae]; *C.albitarsella* Zeller, 1849 [on *Origanumvulgare*: Lamiaceae]; *C.artemisiae* Mühlig. 1864; *C.artemisicolella* Bruand, 1855; *C.chamaedriella* Bruand, 1852; *C.conspicuella* Zeller, 1849 [on *Asterlinosyris*: Asteraceae]; *C.conyzae* Zeller, 1868 [on *Pulicariadysenterica*: Asteraceae]; *C.cracella* (Vallot, 1835); *C.dianthi* Herrich-Schaffer, 1855; *C.follicularis* Vallot, 1802 [on *Pulicariadysenterica*: Asteraceae]; *C.granulatella* Zeller, 1849; *C.inulae* Wocke, 1877 [on *Pulicariadysenterica*: Asteraceae]; *C.linosyridella* Fuchs, 1880; *C.meridionella* Rebel, 1912; *C.salicorniae* Heinemann & Wocke, 1877; *C.salinella* Stainton, 1859; *C.vestianella* (Linnaeus, 1758). **Gelechiidae**: *Aproaeremaanthyllidella* (Hübner, 1813) [on *Anthyllis* sp.: Fabaceae]; *Caryocolumsaginella* (Zeller, 1868); *Chrysoesthiadrurella* (Fabricius, 1775); *Ch.sexguttella* (Thunberg, 1794) [on *Chenopodiumalbum*: Amaranthaceae]; *Scrobipalpaatriplicella* (Fischer v. Röslerstamm, 1841) [on *Chenopodiumalbum*: Amaranthaceae]; *S.gallicella* (Constant, 1885); *S.ocellatella* (Boyd, 1858); *Thiotrichasubocellea* (Stephens, 1834); *Tutaabsoluta* (Meyrick, 1917) [on *Solanumnigrum*: Solanaceae]. **Gracillariidae**: *Micrurapteryxbaranchikovi* sp. nov. [on *Thermopsislanceolata*: Fabaceae] (**new record**). **Heliodinidae**: *Heliodinesroesella* (Linnaeus, 1758) [on *Atriplex* sp.: Amaranthaceae]. **Epermeniidae**: *Ochromolopisictella* (Hübner, 1813). **Tortricidae**: *Olethreutesarbutella* (Linnaeus, 1758); *Spilonotaocellana* (Denis & Schiffermüller, 1775).

####### Distribution.

(according to Yu et al. 2016; Belokobylskij et al. 2019). Russia: Moscow Province, Perm Territory, Altai Territory, Krasnoyarsk Territory, the Republic of Khakassia (**new record**), Irkutsk Province, Zabaikalskiy Territory. Tunisia, Western and Central Europe, Armenia, Turkey, Iran, Kazakhstan, Uzbekistan, Tajikistan, Mongolia, Korea.

####### Remarks.

The species from the Republic of Khakassia has been DNA barcoded for the first time. Given reliable species identification on morphology, the obtained sequence (process ID GPRU034-21) can be used as a reference DNA barcode for molecular-based identification of *Agathisfuscipennis*.

#### Subfamily Microgastrinae Foerster, 1863

##### Genus *Illidops* Mason, 1981

###### Illidops subversor

Taxon classificationAnimaliaHymenopteraBraconidae

(Tobias & Kotenko, 1986)

68DA4077-34E6-52F4-9BF5-B8B9E9720DE5

Apanteles subversor Tobias & Kotenko, 1986: 422; Yu et al. 2016.
Illidops subversor (Tobias & Kotenko, 1986): Kotenko 2007: 178; Belokobylskij et al. 2019: 296.

####### Material examined.

1♀, Republic of Khakassia, near Black Lake field station of SIF SB RAS, 27.VII.2020 coll. (mine), 10.VIII.2020 em., Kirichenko N. coll, no. BL-17-20, host: *Micrurapteryx* sp. nov. (pupa), plant: *Thermopsislanceolata*, Kirichenko N. det.; 1♀, same labels, but 28.VII.2020 coll. (mine), 12.VIII.2020 em., no. BL-4-2; 1♂, same labels, but “7.VII.2020 coll. (mine), 18–23.VII.2020 em., no BL-0-20. All deposited in ZISP.

**Figure 11.**
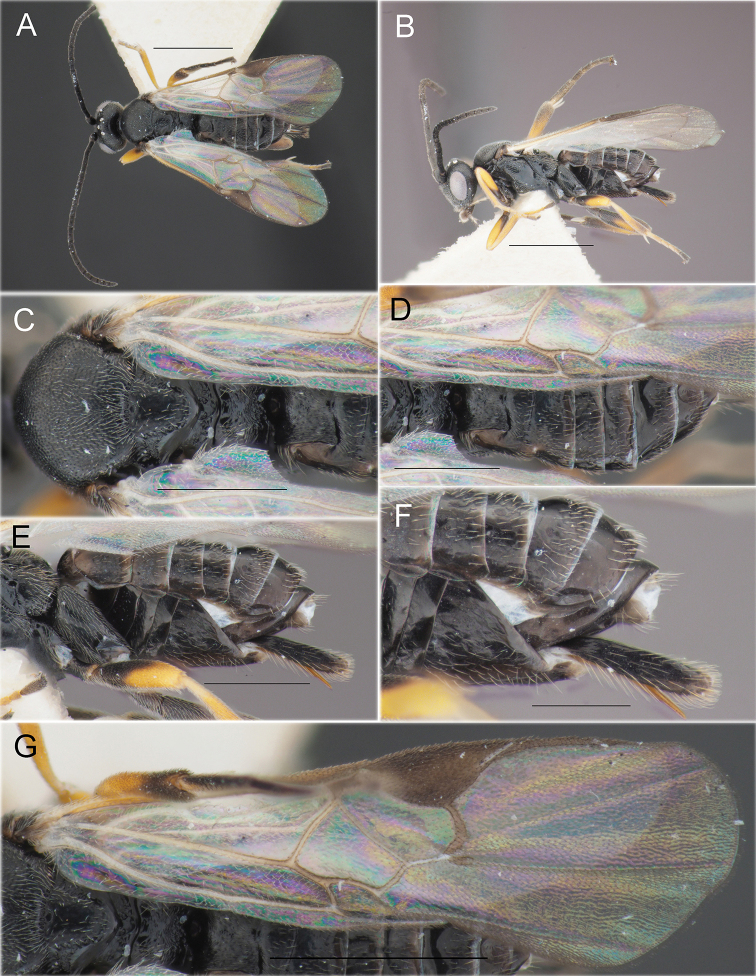
*Illidopssubversor* (Tobias & Kotenko, 1986) (♀, reared specimen, 28.VII.2020 (mine) coll., 12.VIII.2020 em., no. BL-4-20, host: *Micrurapteryxbaranchikovi* sp. nov., the Republic of Khakassia, Russia) **A** habitus, dorsal view **B** habitus, lateral view **C** mesosoma, dorsal view **D** metasoma, dorsal view **E** metasoma, lateral view **F** apical part of metasoma and ovipositor, lateral view **G** fore wing. Scale bars: 1 mm (**A, B, G**), 0.5 mm (**C–E**), 0.3 mm (**F**).

####### Hosts.

**Gracillariidae**: *Micrurapteryxbaranchikovi* sp. nov. [on *Thermopsislanceolata* Brown: Fabaceae] (**new record**).

####### Distribution.

(according to Belokobylskij et al. 2019). Russia: Novosibirsk Province, the Republic of Khakassia (**new record**).

####### Remarks.

The specimens from Khakassia are very similar to the only known specimen, the holotype of *I.subversor* from the south of Western Siberia. However, in the holotype, the pterostigma is irregularly coloured, yellowish-brown medially and dark brown marginally, whereas in reared specimens of *I.subversor* from Khakassia, the pterostigma is mainly dark brown to almost black, but brownish yellow in a small basal spot (Fig. 11G). Such variation in pterostigma colour is known in parasitoids and may be related to specimen preservation.

#### Family Ichneumonidae Latreille, 1802

**Subfamily Campopleginae Foerster, 1869**

##### Genus *Campoplex* Gravenhorst, 1829

###### Campoplex sp. aff. borealis

Taxon classificationAnimaliaHymenopteraIchneumonidae

(Zetterstedt, 1838)

1AC26EFF-E916-5F2C-84D8-99C9870A1623

####### Material examined.

1♀, Republic of Khakassia, near Black Lake field station of SIF SB RAS, along the lake bank, 28.VII.2020 coll. (mine), 14.VIII.2020 em., Kirichenko N. coll., no. BL-1-20-1 (legs taken for DNA), host: *Micrurapteryx* sp. nov. (pupa), plant: *Thermopsislanceolata*; Kirichenko N. det.; 1♀, same labels, but 15.VIII.2020 em., no. BL-3-20; 1♂, same labels, but 13–17.VIII.2020 em., no BL-11-20; 1♀, same labels, but 17.VIII.2020 em., no. BL-5-20; 1♂, same labels, but 18–21.VIII.2020 em., no. BL-9-20; 1♂, same labels, but VII.2020 coll. (mine), 11–16.VIII.2020 em., no. BL-7-20-1 (legs taken for DNA). All deposited in ZISP.

**Figure 12.**
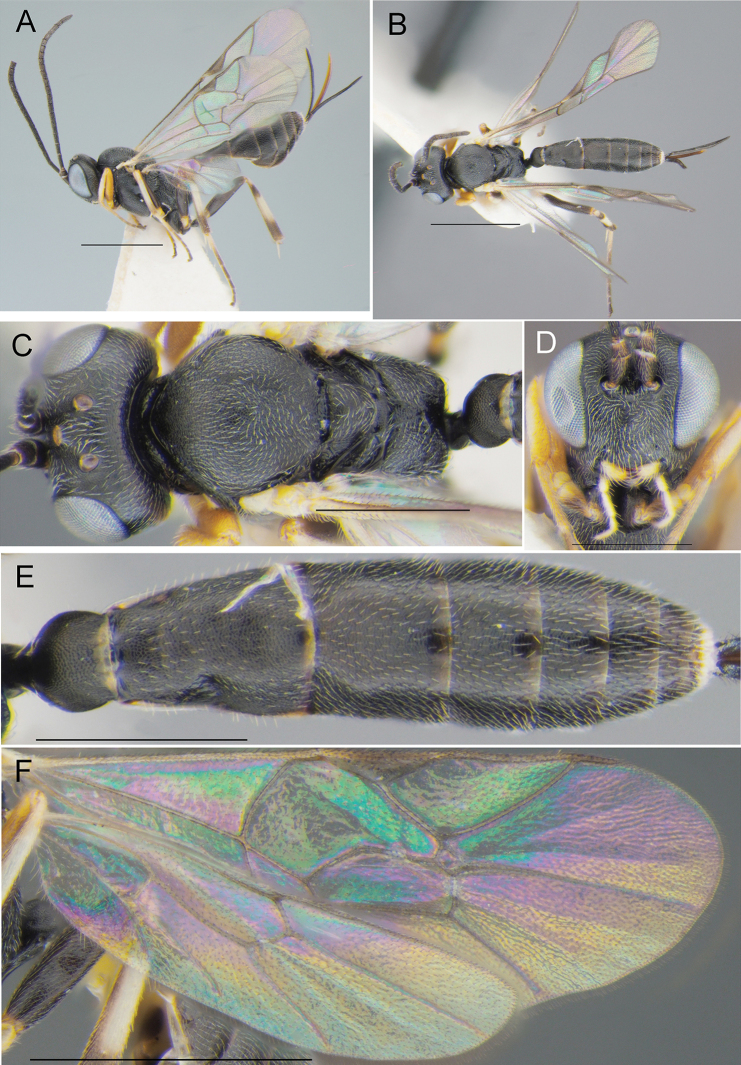
Campoplexsp. aff.borealis (Zetterstedt, 1838) (♀, reared specimen, 28.VII.2020 (mine) coll., 15.VIII.2020 em., no. BL-3-20, host: *Micrurapteryxbaranchikovi*, the Republic of Khakassia, Russia) **A** habitus, lateral view **B** habitus, dorsal view **C** head and mesosoma, dorsal view **D** head, front view **E** metasoma, dorsal view **F** wings. Scale bars: 1 mm (**A, B, F**), 0.5 mm (**C–E**).

####### Hosts.

*Micrurapteryxbaranchikovi* sp. nov. (Gracillariidae).

####### Distribution.

Republic of Khakassia.

####### Remarks.

Morphologically, the specimens of Campoplexsp. aff.borealis from Khakassia are very similar to *Campoplexborealis* from the Western Palearctic (Zetterstedt, 1838) (Andrey I. Khalaim det.), with hind femur dark reddish brown to black, as was already recorded in the specimens from north-west of European Russia (Yu et al. 2016). However, DNA barcoding highlights a significant divergence (i.e., 7.3%) between the Khakassian specimens and *C.borealis* identified from Germany (Fig. 3, Suppl. material 3: Table S3). Such a divergence may suggest the presence of a cryptic species in Khakassia. To test this hypothesis, a special study, involving more sampling across the distributional range of *C.borealis*, would be needed.

## Discussion

Based on integrative taxonomy, we discovered a new species of gracillariid in the Republic of Khakassia (Siberia, Russia) that is described here as *Micrurapteryxbaranchikovi* sp. nov. We showed that morphologically the male of the new species is somewhat similar to the Central Asian *M.sophorivora* feeding on *Sophora* (Fabaceae), whereas the female shows similarity to the North American *M.salicifoliella* feeding on *Salix* and *Populus* (Salicaceae). We highlighted the morphological characters differentiating *M.baranchikovi* from “*Parectopa*” *thermopsella* described in the XIX century in Colorado (USA) feeding on *Thermopsis* (Chambers 1875). Genetically, the new species is divergent from studied representatives of the genus *Micrurapteryx*, with 9.3% of interspecific divergence to the nearest neighbour *M.kollariella*. Morphologically, the latter is significantly different from *M.baranchikovi*. Furthermore, it feeds on a variety of Genisteae legumes, except *Thermopsis* (the tribe Sophoreae) (De Prins and De Prins 2021). The relatedness of *M.baranchikovi* to *Sophora*-feeding *Micrurapteryx* could be explained by the host plant phylogeny: *Thermopsis* (the host plant of *M.baranchikovi*) and *Sophora* (the host of *M.sophorivora*) are from one tribe Sophoreae, whereas Genisteae, on which *Micrurapteryxkollariella* feeds, is a genetically divergent tribe (Cardoso et al. 2013). This hypothesis will need further exploration for which molecular data of all Fabaceae-feeding *Micrurapteryx* would be required.

The new species has the ability to form outbreaks on *Thermopsislanceolata*, as we documented for the Republic of Khakassia in 2020. This plant is harvested for medical purpose in some regions of Russia, including the Republic of Khakassia (Telyatiev 1985). For that purpose, all parts of the plant (except roots) are collected: green parts are harvested during the flowering period in June-July, whereas seeds are collected in August-September (Telyatiev 1985). As we showed in our study, the major damage caused by larvae of *M.baranchikovi* occurs in late July – mid August, which coincides with the harvesting period. The plants with damaged brownish leaves are not collected (Volynskiy et al. 1978). The natural population of *T.lanceolata* have declined due to exhaustive harvesting (Minaeva 1991), and the new moth may potentially affect the populations of this beneficial medical plant in the studied region.

In 2020, we documented a surprisingly low parasitism rate in the dense population of *M.baranchikovi*: only 9% of the pupae were infested by parasitoids. Furthermore, only three parasitoid species were found to attack the new moth species. Notably, before our study both braconids, *Agathisfuscipennis* and *Illidopssubversor*, and the ichneumonid, Campoplexsp. aff.borealis, all reared from *M.baranchikovi* pupae, have never been reported as parasitoids of Gracillariidae.

In general, parasitoids associated with gracillariids are diverse and can be highly abundant; moreover, many parasitoid species are shared by different gracillariid species. For example, the parasitoid complex of the poplar leaf miner, *Phyllonorycterpopulifoliella*, a native outbreaking species in Russia, counts at least 68 species (Ermolaev 2019). In some years, the parasitoids are able to kill up to 77% of the moth’s larvae and pupae (Belova 1994). Many of these native parasitoid species were recruited by the East Asian *Phyllonorycterissikii* (Kumata, 1963) during its invasion to European Russia (Ermolaev et al. 2018).

So far, parasitoids have been known only for two *Micrurapteryx* species: *M.sophorivora* and *M.kollariella* (De Prins and De Prins 2021). As documented in Turkey, *M.sophorivora* is attacked exclusively by chalcidoids: *Baryscapusnigroviolaceus* (Nees, 1834), *Cirrospiluspictus* (Nees, 1834), *Necremnuscroton* (Walker, 1839), *Neochrysocharisarvensis* Graham, 1963, *N.formosus* (Westwood, 1833), *Pnigalio* sp. (all from family Eulophidae) and *Pteromalus* sp. (Pteromalidae) (Gençer and Seven 2005). In Europe, for *M.kollariella* three parasitoids were recorded: one eulophid, *Pnigalioagraules* (Walker, 1839) and two ichneumonids, *Diadegmaholopyga* (Thomson, 1887) and *Scambusannulatus* (Kiss, 1924) (Vidal and Buszko 1990; Sawoniewicz and Buszko 1994). The majority of these parasitoid species are also known in Europe on various gracillariids, in particular on the representatives of five subfamilies: *Cameraria*, *Phyllonorycter* (Lithocolletinae), *Caloptilia*, *Gracillaria* (Gracillariinae), *Parornix* (Parornichinae), *Metriochroa* (Oecophyllembiinae), and *Phyllocnistis* (Phyllocnistinae) (Kawahara et al. 2016). Surprisingly, in our study we found none of these species parasitising *M.baranchikovi*, despite that some occur in Russia (Belokobylskij et al. 2019).

Our study highlights the complexity of identifying the species of *Campoplex* from the Republic of Khakassia based on both morphology and DNA barcoding. Representatives of Campopleginae are still very scarcely studied in Russia (Belokobylskij et al. 2019). As a result, the Khakassian specimens are only preliminary determined here as Campoplexsp. aff.borealis. Furthermore, we noticed a mismatch in *C.multicinctus* identification in BOLD, and we suspect misidentification of one Finnish specimen of *C.multicinctus* on this platform that turned to be highly similar genetically to the Khakassian *C*. sp. aff.borealis. However, the latter morphologically and genetically is more similar to *C.borealis*, as we have shown in the study. We do not exclude that the Siberian *C*. sp. aff.borealis may represent a cryptic species.

Further study would be needed to define the range of the new moth and assess its potential impact on *T.lanceolata*, as well as to explore the complex of parasitoids associated with *M.baranchikovi* in Siberia and clarify their intraspecific divergence at morphological and molecular genetic levels.

## Supplementary Material

XML Treatment for Micrurapteryx baranchikovi

XML Treatment for Agathis fuscipennis

XML Treatment for Illidops subversor

XML Treatment for Campoplex sp. aff. borealis

## Supplementary materials

Supplementary material 1 Table S1 Data type Specimen data Explanation note The list of specimens of *Micrurapteryx* and parasitoid specied involved into molecular genetic analysis. https://binary.pensoft.net/file/597139This dataset is made available under the Open Database License (http://opendatacommons.org/licenses/odbl/1.0/). The Open Database License (ODbL) is a license agreement intended to allow users to freely share, modify, and use this Dataset while maintaining this same freedom for others, provided that the original source and author(s) are credited.Natalia I. Kirichenko, Evgeny N. Akulov, Paolo Triberti, Sergey A. Belokobylskij

Supplementary material 2 Table S2 Data type Genetic data Explanation note The list of diagnostic substitutions in COI mtDNA gene in *Micrurapteryxbaranchikovi* sp. nov. and *M.kollariella*. https://binary.pensoft.net/file/597140This dataset is made available under the Open Database License (http://opendatacommons.org/licenses/odbl/1.0/). The Open Database License (ODbL) is a license agreement intended to allow users to freely share, modify, and use this Dataset while maintaining this same freedom for others, provided that the original source and author(s) are credited.Natalia I. Kirichenko, Evgeny N. Akulov, Paolo Triberti, Sergey A. Belokobylskij

Supplementary material 3 Table S3 Data type Genetic data Explanation note Genetic divergences in COI mtDNA gene in studied *Campoplex* parasitoids. https://binary.pensoft.net/file/597141This dataset is made available under the Open Database License (http://opendatacommons.org/licenses/odbl/1.0/). The Open Database License (ODbL) is a license agreement intended to allow users to freely share, modify, and use this Dataset while maintaining this same freedom for others, provided that the original source and author(s) are credited.Natalia I. Kirichenko, Evgeny N. Akulov, Paolo Triberti, Sergey A. Belokobylskij

## References

[B1] Bai HY (2013) A new species of *Micrurapteryx* Spuler (Lepidoptera: Gracillariidae) from Tibet, China. Entomological News 122(4): 324–327. https://doi.org/10.3157/021.122.0404

[B2] Belokobylskij SA Samartsev KG Il’inskaya AS [Eds] (2019) Annotated catalogue of the Hymenoptera of Russia. Volume II. Apocrita: Parasitica. Proceedings of the Zoological Institute Russian Academy of Sciences. Supplement 8. Zoological Institute RAS, St. Petersburg, 594 pp. https://doi.org/10.31610/trudyzin/2019.supl.8.5

[B3] Belova NK (1994) Pests of urban green spaces. Zashchita rasteniy (Plant protection) 8: 37–38. [In Russian]

[B4] Braun AF (1925) Microlepidoptera of northern Utah. Transactions of the American Entomological Society 51: 183–226.

[B5] Cardoso D Pennington RT de Queiroz LP Boatwright JS Van Wyk B-E Wojciechowski MF Lavin M (2013) Reconstructing the deep-branching relationships of the papilionoid legumes. South African Journal of Botany 89: 58–75. https://doi.org/10.1016/j.sajb.2013.05.001

[B6] Chambers VT (1875) Teneina [sic] of Colorado. The Cincinnati Quarterly Journal of Science 2(4): 289–305.

[B7] Davis DR (1983) Gracillariidae. In: Hodges RW (Ed.) Check list of the Lepidoptera of America North of Mexico including Greenland.EW Classey Ltd., Faringdon, and The Wedge Entomological Research Foundation, Washington, 9–11.

[B8] De Prins J De Prins W (2021) Global Taxonomic Database of Gracillariidae (Lepidoptera). http://www.gracillariidae.net

[B9] de Waard JR Ivanova NV Hajibabaei M Hebert PDN (2008) Assembling DNA barcodes: analytical methods. In: Cristopher M (Ed.) Methods in molecular biology: environmental genetics.Humana Press Inc., Totowa, USA, 275–293. https://doi.org/10.1007/978-1-59745-548-0_15

[B10] Eiseman C (2019) Leafminers of North America. Self-published e-book, clii + 1857 pp. [*Thermopsis*, 807–808.]

[B11] Ermolaev IV (2019) Ecological mechanisms of nonperiodical population wave: a case study of the poplar leafminer – *Phyllonorycterpopulifoliella* (Lepidoptera, Gracillariidae). Zhurnal Obshchei Biologii (Journal of General Biology) 80(6): 451–476. [In Russian] https://doi.org/10.1134/S0044459619060034

[B12] Ermolaev IV Yefremova ZA Domrachev TB (2018) The influence of parasitoids (Hymenoptera, Eulophidae) on the survival of the lime leafminer (*Phyllonorycterissikii*, Lepidoptera, Gracillariidae) in Udmurtia. Zoologicheskii Zhurnal (Zoological Journal) 97(4): 401–407. [In Russian] https://doi.org/10.1134/S0013873818040024

[B13] Gençer L Seven S (2005) Chalcidoid parasitoids of *Micrurapteryxsophorivora* (Lepidoptera: Gracillariidae) in Kuluncak, Turkey. Phytoprotection 86: 133–134. https://doi.org/10.7202/012513ar

[B14] Grigoryev AA Budyko MA (1960) Classification of the Climates of the USSR. Soviet Geography 1: 3–24. [In Russian] https://doi.org/10.1080/00385417.1960.10769843

[B15] Hajek P McRobbie H Myers K (2013) Efficacy of cytisine in helping smokers quit: systematic review and meta-analysis. Thorax 68(11): 1037–1042. https://doi.org/10.1136/thoraxjnl-2012-203035 23404838 10.1136/thoraxjnl-2012-203035

[B16] Hebert PDN Ratnasingham S Zakharov EV Telfer AC Levesque-Beaudin V Milton MA Pedersen S Jannetta P deWaard JR (2016) Counting animal species with DNA barcodes: Canadian insects. Philosophical Transactions of the Royal Society B 371: 20150333. https://doi.org/10.1098/rstb.2015.0333 10.1098/rstb.2015.0333 PMC4971185 27481785

[B17] Jeanmougin F Thompson JD Gouy M Higgins DG Gibson TJ (1998) Multiple sequence alignment with Clustal X. Trends in Biochemical Sciences 23: 403–405. https://doi.org/10.1016/S0968-0004(98)01285-7 9810230 10.1016/s0968-0004(98)01285-7

[B18] Kawahara AY Plotkin D Ohshima I Lopez-Vaamonde C Houlihan PR Breinholt JW Kawakita A Xiao L Regier JC Davis DR Kumata T Sohn JC De Prins J Mitter C (2017) A molecular phylogeny and revised higher-level classification for the leaf-mining moth family Gracillariidae and its implications for larval host-use evolution. Systematic Entomology 42(1): 60–81. https://doi.org/10.1111/syen.12210

[B19] Kirichenko N Triberti P Mutanen M Magnoux E Landry J-F Lopez-Vaamonde C (2016) Systematics and biology of some species of *Micrurapteryx* Spuler (Lepidoptera, Gracillariidae) from the Holarctic Region, with re-description of *M.caraganella* (Hering) from Siberia. ZooKeys 579: 99–156. https://doi.org/10.3897/zookeys.579.7166 10.3897/zookeys.579.7166 PMC4829971 27110203

[B20] Klots A (1970) Lepidoptera. In: Tuxen SL (Ed.) Taxonomist’s glossary of genitalia in insects.Munksgaard, Copenhagen, 115–139.

[B21] Kotenko AG (2007) Subfam. Microgastrinae. In: Lelej AS (Ed.) Key to insects of the Russian Far East.Vol. IV. Neuropteroidea, Mecoptera, Hymenoptera. Pt 5, Dalnauka, Vladivostok, 134–192. [In Russian]

[B22] Kotunkov GN (1974) The cultivated and wild medical plants: the handbook. Naukova Dumka, Kiev, 174 pp. [In Russian]

[B23] Kristensen NP (2003) Skeleton and muscles: adults. In: Kristensen NP (Ed.) Lepidoptera, Moths and Butterflies (Volume 2) – Morphology, physiology, and development.Handbook of Zoology 36(4), Walter de Gruyter, Berlin, 39–131. https://doi.org/10.1515/9783110893724.39

[B24] Kumar S Stecher G Li M Knyaz C Tamura K (2018) MEGA X: Molecular evolutionary genetics analysis across computing platforms. Molecular Biology and Evolution 35: 1547–1549. https://doi.org/10.1093/molbev/msy096 29722887 10.1093/molbev/msy096 PMC5967553

[B25] Kuznetzov VI Tristan NI (1985) A review of the leaf blotch miners of the genus *Micrurapteryx* Spuler (LepidopteraGracillariidae) of the Palaearctic fauna. Entomologicheskoe Obozrenie 64(1): 177–192. [In Russian]

[B26] Lager AA (1988) Phytotherapy. Krasnoyarsk University, Krasnoyarsk, 272 pp. [In Russian]

[B27] McDunnough J (1939) Check list of the Lepidoptera of Canada and the United States of America. Part II. Microlepidoptera. Memoirs of the Southern California Academy of Sciences 2(1): 1–171.

[B28] Meyer CP Paulay G (2005) DNA barcoding: Error rates based on comprehensive sampling. PLoS Biology 3: 2229–2238. https://doi.org/10.1371/journal.pbio.0030422 10.1371/journal.pbio.0030422 PMC1287506 16336051

[B29] Minaeva VG (1991) The medical plants of Siberia. Nauka, Siberian branch, Novosibirsk, 431 pp. [In Russian]

[B30] Noreika R Puplesis R (1992) Description of new species of moths of the family Gracillariidae (Lepidoptera) from Azerbaijan and Middle Asia and synonymy of *Gracillariaimpictipennella* Grsm. Entomological Review 74(5): 43–51.

[B31] Ratnasingham S Hebert PDN (2007) BOLD: The Barcode of Life Data System (http://www.barcodinglife.org). Molecular Ecology Notes 7: 355–364. https://doi.org/10.1111/j.1471-8286.2007.01678.x 10.1111/j.1471-8286.2007.01678.x PMC1890991 18784790

[B32] Ratnasingham S Hebert PDN (2013) A DNA-based registry for all animal species: the barcode index number (BIN) system. PLoS ONE 8(8): e66213. https://doi.org/10.1371/journal.pone.0066213 10.1371/journal.pone.0066213 PMC3704603 23861743

[B33] Robinson GS (1976) The preparation of slides of Lepidoptera genitalia with special reference to the Microlepidoptera. Entomologist´s Gazette 27: 127–132.

[B34] Samoylova GS Goryachko MD Kyzlasov IL Torbostaev KM Goryachko MD Prokinova AN Seleverstov VV Kyzlasov IL (2019) Khakassia. Nature: climate. In: Kravets SL (Ed.) The Great Russian Encyclopedia. https://bigenc.ru/geography/text/5454659 [In Russian]

[B35] Sawoniewicz J Buszko J (1994) Ichneumonidae (Hymenoptera) reared from mining Lepidoptera in Poland. Wiadomosci Entomologiczne 13(1): 55–61.

[B36] Seven S Genҫer L (2009) Contribution to the biology and distribution of the leaf-mining moth *Micrurapteryxsophorivora* Kuznetzov & Tristan, 1985 (LepidopteraGracillariidae). SHILAP 37: 511–514.

[B37] Simbolotti G van Achterberg C (1999) Revision of the west Palaearctic species of the genus *Agathis* Latreille (Hymenoptera: Braconidae: Agathidinae). Zoologische Verhandelingen Leiden 325: 1–167.

[B38] Telyatiev VV (1985) Useful plants of Central Siberia. East Siberian publishing house, Irkutsk, 384 pp. [In Russian]

[B39] Tobias VI Belokobylskij SA Kotenko AG (1986) Fam. Braconidae. In: Medvedev GS (Ed.) Opredelitel Nasekomych Evrospeyskoy Chasti SSSR 3, Pereponchatokrylye 4. [Keys to insects of the European part of USSR. Hymenoptera.]. Nauka, Leningrad, 500 pp. [In Russian]

[B40] Tolmachev AI [Ed.] (1974) The key to the plants of Yakutia. Nauka, Novosibirsk, 543 pp. [In Russian]

[B41] Vári L (1961) South African Lepidoptera (Vol. I) – Lithocolletidae. Transvaal Museum Memoir 12: 1–238.

[B42] Vidal Handbook: Medical drugs in Russia (2021) Cough tablets (Antitussive pills): instructions for use. https://www.vidal.ru/drugs/antitussive_tablets__30376 [In Russian]

[B43] Vidal S Buszko J (1990) Studies on the mining Lepidoptera of Poland. VIII. Chalcidoid wasps reared from mining Lepidoptera (Hymenoptera, Chalcidoidea). Polskie Pismo Entomologiczne 60: 73–103.

[B44] Vieira V Karsholt O (2010) Lepidoptera, 219–221. In: Borges PAV, Costa A, Cunha R, Gabriel R, Gonçalves V, Martins AF, Melo I, Parente M, Raposeiro P, Rodrigues P, Santos RS, Silva L, Vieira P, Vieira V (Eds) A list of the terrestrial and marine biota from the Azores, Princípia, Cascais, 432 pp.

[B45] Volynskiy BG Bender KI Freidman SL Bogoslovskaya SI Voronina KV Glazyrina GA Kaprelova TS Koloskova IG Kuznetsova IG Martynov LA (1978) Medical plants in traditional and folk medicine. 5^th^ edn. Saratov University, Saratov, 360 pp. [In Russian]

[B46] West R Zatonski W Cedzynska M Lewandowska D Pazik J Aveyard P Stapleton J (2011) Placebo-controlled trial of cytisine for smoking cessation. New England Journal of Medicine 365(13): 1193–1200. https://doi.org/10.1056/NEJMoa1102035 10.1056/NEJMoa1102035 21991893

[B47] Wu Z Raven PH [Eds] (2010) Flora of China. Beijing, Science Press, St. Louis, Missouri Botanical Garden Press 10: 1–642.

[B48] Yu DSK van Achterberg C Horstmann K (2016) Taxapad 2016, Ichneumonoidea 2015. Database on flash-drive (CD). www.taxapad.com

[B49] Zhu X Kirkbride Jr JH (2005) Proposal to conserve the names *Thermopsislanceolata* and *Sophoralupinoides* with a conserved type (Leguminosae). Taxon 55(4): 1047–1049. https://doi.org/10.2307/25065715

