# Supplementary material for: A new species of Micrurapteryx (Lepidoptera, Gracillariidae) feeding on Thermopsislanceolata (Fabaceae) in southern Siberia and its hymenopterous parasitoids
Source: Zookeys. 2021 Oct 8;1061:131–63. doi: 10.3897/zookeys.1061.70929 (PMC8520033; doi:10.3897/zookeys.1061.70929)
Supplement: Supplementary material 1 — Table S1 [file zookeys-1061-131-s001.docx]

**Authors:** Natalia I. Kirichenko, Evgeny N. Akulov, Paolo Triberti, Sergey A. Belokobylskij

**Title:** A new species of *Micrurapteryx* (Lepidoptera: Gracillariidae) feeding on *Thermopsis lanceolata* (Fabaceae) in southern Siberia and its hymenopterous parasitoids

**Table S1.** The specimens of *Micrurapteryx* and parasitoid species involved in the molecular genetic analysis. For each specimen, Sample ID and process ID are provided linking the records in the BOLD database with the voucher specimen data.

| **№** | **Sample ID*, life stage [L – larva, P – pupa, A – adult]** | **Process ID** | **Host** | **Country** | **Region^**^** | **Collection date** | **Collector** | **GenBank**  **Accession** |
| --- | --- | --- | --- | --- | --- | --- | --- | --- |
| **GRACILLARIIDAE** | | | | | | | | |
| *Micrurapteryx baranchikovi* sp. nov. | | | | | | | | |
| 1 | NK805, (EA-BL-1) [L] | LMINH181-19 | *Thermopsis lanceolata* | Russia | KhRep | 31.VII.2019 | E. Akulov, N. Kirichenko | MZ661077 |
| 2 | NK-20-15,  (NK-08.12-1, ♂, holotype) [A] | GPRU015-21 | idem | Russia | KhRep | 28.VII.2020 | N. Kirichenko | MZ661071 |
| 3 | NK-20-16,  (NK-29.11-1, ♀, paratype), genit. slide ) [A] | GPRU016-21 | idem | Russia | KhRep | 28.VII.2020 | N. Kirichenko | MZ661079 |
| 4 | NK-20-17,  (NK-08.12-2, ♀, paratype) [A] | GPRU017-21 | idem | Russia | KhRep | 28.VII.2020 | N. Kirichenko | MZ661072 |
| 5 | NK-20-18,  (NK-26.11-1, ♀, paratype) [A] | GPRU018-21 | idem | Russia | KhRep | 28.VII.2020 | N. Kirichenko | MZ661075 |
| 6 | VZ-20-10,  (Kh-NK-20-1) [L] | GPRU044-21 | idem | Russia | KhRep | 28.VII.2020 | N. Kirichenko | MZ661073 |
| 7 | VZ-20-11,  (Kh-NK-20-2) [L] | GPRU045-21 | idem | Russia | KhRep | 27.VII.2020 | N. Kirichenko | MZ661078 |
| *Micrurapteryx kollariella* (Zeller, 1839) | | | | | | | | |
| 8 | CLV5200 [A] | LNOUD2104-12 | ― | Italy | ER | 20.VII.1993 | L. Usvelli | KP845417 |
| 9 | TLMF Lep 03534 [A] | PHLAD359-11 | ― | Italy | Pied | 04.V.2008 | P. Huemer | JN272048 |
| 10 | CLV2281 [A] | GRPAL123-11 | ― | France | PACA | 20.V.2005 | A. Cama | KP845406 |
| *Micrurapteryx gradatella* (Herrich-Schäffer, 1855) | | | | | | | | |
| 11 | NK435 [A] | MICRU007-15 | *Lathyrus linifolius* | Finland | Turku | 16.VI.2010 | M. Mutanen | KP845413 |
| 12 | NK436 [A] | MICRU008-15 | idem | Finland | Turku | 16.VI.2010 | M. Mutanen | KP845411 |
| 13 | NK437 [A] | MICRU009-15 | idem | Finland | Turku | 01.VI.2013 | M. Mutanen | KP845403 |
| 14 | NK459 [L] | MICRU014-15 | *Vicia amoena* | Russia | KrT | 03.VII.2015 | N. Kirichenko | KU380248 |
| 15 | NK462 [L] | MICRU017-15 | *V. amoena* | Russia | KrT | 05.VII.2015 | N. Kirichenko | KU380266 |
| 16 | NK471 [L] | MICRU026-15 | idem | Russia | KrT | 05.VII.2015 | N. Kirichenko | KU380245 |
| 17 | NK589 [L] | SIBLE078-17 | *Vicia sp.* | Russia | AmP | 26.VI.2016 | N. Kirichenko | MK403682 |
| 18 | NK614 [L] | SIBLE103-18 | *V. unijuga* | Russia | KrT | 09.VIII.2017 | N. Kirichenko | MK029652 |
| *Micrurapteryx occulta* (Braun, 1922) | | | | | | | | |
| 19 | BIOUG02884-D02 [A] | CNJAA025-12 | ― | Canada | AB | 30.V.2012 | B. Sharp, C. Syfchuk | KM540469 |
| 20 | CNCLEP00108894 [A] | MNAQ402-15 | ― | Canada | BC | 21.VI.2013 | D.G. Holden | KU380265 |
| 21 | CNCLEP00121159 [A] | MNAQ069-15 | *Lupinus* | Canada | BC | 17.VI.2014 | J.-F. Landry, D. Holden | KU380261 |
| *Micrurapteryx caraganella* (Hering, 1957) | | | | | | | | |
| 22 | NK189 [A] | ISSIK234-14 | *Caragana arborescens* | Russia | KrT | 12.VII.2013 | N. Kirichenko | KP845393 |
| 23 | NK414 [A] | ISSIK363-14 | idem | Russia | KrT | 18.VIII.2014 | N. Kirichenko | KP845397 |
| 24 | NK415 [A] | ISSIK364-14 | idem | Russia | KrT | 18.VIII.2014 | N. Kirichenko | KP845405 |
| 25 | NK416 [A] | ISSIK365-14 | idem | Russia | KrT | 18.VIII.2014 | N. Kirichenko | KP845402 |
| 26 | NK417 [A] | ISSIK366-14 | idem | Russia | KrT | 18.VIII.2014 | N. Kirichenko | KP845424 |
| 27 | NK418 [A] | ISSIK367-14 | idem | Russia | KrT | 18.VIII.2014 | N. Kirichenko | KP845391 |
| 28 | NK429 [P] | MICRU001-15 | idem | Russia | NO | 03.VIII.2011 | N. Kirichenko | KP845418 |
| 29 | NK430 [A] | MICRU002-15 | idem | Russia | KrT | 15.VII.2014 | E. Akulov | KP845400 |
| 30 | NK431 [A] | MICRU003-15 | idem | Russia | KrT | 15.VII.2014 | E. Akulov | KP845415 |
| 31 | NK432 [A] | MICRU004-15 | idem | Russia | KrT | 15.VII.2014 | E. Akulov | KP845389 |
| 32 | NK433 [A] | MICRU005-15 | idem | Russia | NO | 02.VII.2013 | N. Kirichenko | KP845387 |
| 33 | NK434 [A] | MICRU006-15 | idem | Russia | NO | 02.VII.2013 | N. Kirichenko | KP845425 |
| 34 | NK473 [A] | MICRU028-15 | idem | Russia | OmP | 23.VII.2015 | N. Kirichenko | KU380247 |
| 35 | NK474 [L] | MICRU029-15 | idem | Russia | OmP | 23.VII.2015 | N. Kirichenko | KU380268 |
| 36 | NK475 [L] | MICRU030-15 | idem | Russia | TyuP | 24.VII.2015 | N. Kirichenko | KU380254 |
| 37 | NK476 [L] | MICRU031-15 | idem | Russia | TyuP | 25.VII.2015 | N. Kirichenko | KU380246 |
| 38 | NK477 [L] | MICRU032-15 | idem | Russia | AlT | 27.VII.2015 | N. Kirichenko | KU380257 |
| 39 | NK478 [P] | MICRU033-15 | idem | Russia | IrkP | 07.VIII.2015 | N. Kirichenko | KU380267 |
| 40 | NK58 [L] | GRPAL1102-13 | *C. boisii* | Russia | NO | 06.VI.2012 | N. Kirichenko | KP845396 |
| 41 | NK470 [L] | MICRU025-15 | *C. frutex* | Russia | OmP | 23.VII.2015 | N. Kirichenko, | KU380252 |
| 42 | NK472 [L] | MICRU027-15 | *Medicago sativa* | Russia | OmP | 23.VII.2015 | N. Kirichenko | KU380260 |
| *Micrurapteryx salicifoliella* (Chambers, 1872) | | | | | | | | |
| 43 | 10PROBE-25766 [A] | PHLCH349-10 | ― | Canada | MB | 06.VIII.2010 | B. Laforest, P. Hebert | JF860441 |
| 44 | 10BBCLP-2121 [A] | BBLPD123-10 | ― | Canada | SK | 16.VIII.2010 | BIObus 2010 | KM546499 |
| 45 | 10BBCLP-2122 [A] | BBLPD124-10 | ― | Canada | SK | 16.VIII.2010 | BIObus 2010 | KM551613 |
| *Parectopa ononidis* (Zeller, 1839) (outgroup) | | | | | | | | |
| 46 | NK461 [L] | MICRU016-15 | *Trifolium pratense* | Russia | KrT | 05.VII.2015 | N. Kirichenko | KU380258 |
| 47 | NK615 [L] | SIBLE104-18 | *Lupinaster pentaphyllus* | Russia | KrT | 10.VIII.2017 | N. Kirichenko | MK029620 |
| 48 | NK638 [L] | SIBLE127-18 | *Trifolium sp.* | Russia | TomP | 27.VI.2017 | N. Kirichenko | MK029647 |
| **HYMENOPTERAN PARASITOIDS** | | | | | | | | |
| *Campoplex* sp. aff*. borealis* (Zetterstedt, 1838) (Ichneumonidae, Campopleginae) | | | | | | | | |
| 49 | NK-20-33 [A] | GPRU033-21 | *Micrurapteryx baranchikovi* | Russia | KhRep | 28.VII.2020 | N. Kirichenko | MZ661074 |
| 50 | NK-20-32 [A] | GPRU032-21 | idem | Russia | KhRep | 28.VII.2020 | N. Kirichenko | MZ661080 |
| *Campoplex borealis* Zetterstedt 1838 [as identified in BOLD] | | | | | | | | |
| 51 | BIOUG42709-E09 [A] | GMGMP3655-18 | ― | Germany | Bavaria | 05.06.2017 | A. Hausman | MZ711411 |
| *Campoplex multicinctus* Gravenhorst, 1829 [as identified in BOLD] | | | | | | | | |
| 52 | BIOUG16302-E04 [A] | GMNWK1280-14 | ― | Norway | Sor-Tr | 08.03.2014 | E. Stur, T. Ekrem | MZ661081 |
| 53 | FICH-001594 [A] | ICHFI1689-13 | ― | Finland | Karuna | 29.07.1999 | R. Jussila | MZ625928 |
| 54 | FICH-001595 [A] | ICHFI1690-13 | ― | Finland | Karuna | 29.07.1999 | R. Jussila | MZ627955 |
| 55 | BIOUG36683-A07 [A] | GMBML364-17 | ― | Belarus | Minsk | 09.06.2016 | T. Lipinksaya | MZ661082 |
| 56 | BIOUG27836-D08 [A] | GMRUC714-16 | ― | Russia | PT | 12.09.2015 | A.Voronkov | MZ661218 |
| *Campoplex* sp.*** | | | | | | | | |
| 57 | FICH-001596 [A] | ICHFI1691-13 | ― | Finland | Ku | 30.06.1998 | R. Jussila | MZ626604 |
| *Agathis fuscipennis* (Zetterstedt, 1838) (Braconidae, Agathidinae) | | | | | | | | |
| 58 | NK-20-34 [A] | GPRU034-21 | *Micrurapteryx baranchikovi* | Russia | KhRep | 28.VII.2020 | N. Kirichenko | MZ661076 |
| *Agathis* sp. | | | | | | | | |
| 59 | JSHYP392-11 [A] | JSHYP392-11 | ― | Canada | ON | 19.09.2010 | J. Sones | KR791690 |
| *Metallus albipes* (Cameron, 1875) (Tenthredinidae) (outgroup) | | | | | | | | |
| 60 | NK243 [L] | ISSIK107-14 | *Rubus idaeus* | Russia | KrT | 31.VII.2011 | N. Kirichenko | MZ711410 |

* For *M. baranchikovi*, field ID is additionally provided in ( ).

** Region: Canada [AB – Alberta, SK – Saskatchewan, BC – British Columbia, ON – Ontario, MB – Manitoba]; France [PACA – Provence-Alpes-Cote d'Azur, Sis – Sisteron]; Finland [Ku – Kuusamo]; Italy [ER – Emilia-Romagna, Pied – Piedmont]; Norway [Sor-Tr – Sor-Trondelag]; Russia [AlT – Altai Territory, AmP – Amur Province, IrkP – Irkutsk Province, KhRep – Khakassia Republic, KrT – Krasnoyarsk Territory, NP – Novosibirsk Province, OmR – Omsk Region, TomP – Tomsk Province, TyuP – Tyumen Province, PT – Primorsky Territory]**.**

*** Mistakenly identified in BOLD as *Campoplex multicinctus* (see text, section Results / Molecular data / Hymenopterous parasitoids).
