# Supplementary material for: A new species of Micrurapteryx (Lepidoptera, Gracillariidae) feeding on Thermopsislanceolata (Fabaceae) in southern Siberia and its hymenopterous parasitoids
Source: Zookeys. 2021 Oct 8;1061:131–63. doi: 10.3897/zookeys.1061.70929 (PMC8520033; doi:10.3897/zookeys.1061.70929)
Supplement: Supplementary material 2 — Table S2 [file zookeys-1061-131-s002.docx]

**Authors:** Natalia I. Kirichenko, Evgeny N. Akulov, Paolo Triberti, Sergey A. Belokobylskij

**Title:** A new species of *Micrurapteryx* (Lepidoptera: Gracillariidae) feeding on *Thermopsis lanceolata* (Fabaceae) in southern Siberia and its hymenopterous parasitoids

**Table S2.** Diagnostic substitutions in COI mtDNA gene in *Micrurapteryx baranchikovi* sp. nov. versus *M. kollariella*.

| Species | Nucleotide position | | | | | | | | | | | |
| --- | --- | --- | --- | --- | --- | --- | --- | --- | --- | --- | --- | --- |
|  | **24** | **28** | **30** | **37** | **45** | **64** | **66** | **69** | **81** | **84** | **96** | **117** |
| *M. baranchikovi* sp. nov. | T | A | T | T | A | C | T | A | A | G | C | A |
| *M. kollariella* | A | G | A | A | T | T | A | T | T | A | T | C |
|  | Nucleotide position | | | | | | | | | | | |
|  | **153** | **168** | **210** | **218** | **240** | **261** | **264** | **267** | **271** | **285** | **327** | **330** |
| *M. baranchikovi* sp. nov. | T | C | T | A | T | C | A | A | T | A | T | T |
| *M. kollariella* | A | T | C | G | C | A | T | T | C | C | G | C |
|  | Nucleotide position | | | | | | | | | | | |
|  | **339** | **357** | **369** | **381** | **391** | **393** | **397** | **399** | **402** | **411** | **423** | **432** |
| *M. baranchikovi* sp. nov. | C | T | C | A | T | A | T | A | T | T | G | C |
| *M. kollariella* | T | C | T | C | C | T | C | T | A | A | A | T |
|  | Nucleotide position | | | | | | | | | | | |
|  | **450** | **498** | **504** | **513** | **516** | **520** | **528** | **532** | **534** | **537** | **538** | **543** |
| *M. baranchikovi* sp. nov. | T | T | A | C | T | T | C | T | A | T | T | T |
| *M. kollariella* | C | A | T | T | A | C | T | C | T | C | C | C |
|  | Nucleotide position | | | | | | | | | | | |
|  | **546** | **547** | **555** | **597** | **606** | **609** | **612** | **615** | **618** | **621** | **634** | **―** |
| *M. baranchikovi* sp. nov. | A | T | A | T | C | G | T | A | A | G | C | ― |
| *M. kollariella* | T | C | T | C | T | T | A | T | G | A | T | ― |
