# Supplementary material for: A new species of Micrurapteryx (Lepidoptera, Gracillariidae) feeding on Thermopsislanceolata (Fabaceae) in southern Siberia and its hymenopterous parasitoids
Source: Zookeys. 2021 Oct 8;1061:131–63. doi: 10.3897/zookeys.1061.70929 (PMC8520033; doi:10.3897/zookeys.1061.70929)
Supplement: Supplementary material 3 — Table S3 [file zookeys-1061-131-s003.docx]

**Authors:** Natalia I. Kirichenko, Evgeny N. Akulov, Paolo Triberti, Sergey A. Belokobylskij

**Title:** A new species of *Micrurapteryx* (Lepidoptera: Gracillariidae) feeding on *Thermopsis lanceolata* (Fabaceae) in southern Siberia and its hymenopterous parasitoids

**Table S3.** Intra- and interspecific divergences in COI mtDNA gene in studied *Campoplex* parasitoids. Minimal pairwise distances are given for each species pair; values in square brackets represent maximal intraspecific distances.

| Species | *Campoplex* sp. aff. *borealis* \|reared from *M. baranchikovi\|* (Russia, Khakassia) | *Campoplex borealis* (Germany) [as identified in BOLD] | *Campoplex multicinctus* [as identified in BOLD]* |
| --- | --- | --- | --- |
| *Campoplex* sp. aff. *borealis*\|reared from *M.  baranchikovi\|* (Russia, Khakassia) | [0] |  |  |
| *Campoplex borealis* (Germany) [as identified in BOLD] | 7.16* | [―]** |  |
| *Campoplex multicinctus* [as identified in BOLD] | 11.61 | 11.03 | [0.39]*** |

* The “true” intraspecific molecular genetic divergence in *Campoplex borealis* needs to be verified on a large sampling set;

** Impossible to assess because sampling was represented by a single individual;

*** The sequences used in this analysis are listed in Table S1. The doubtful sequence of *Campoplex multicinctus* (Finland, sample ID: FICH-001596, process ID: ICHFI1691-13, R. Jussila coll. & det.) was not included in this analysis.
